# Premature Neural Progenitor Cell Differentiation Into Astrocytes in Retinoic Acid-Induced Spina Bifida Rat Model

**DOI:** 10.3389/fnmol.2022.888351

**Published:** 2022-06-17

**Authors:** Marc Oria, Bedika Pathak, Zhen Li, Kenan Bakri, Kara Gouwens, Maria Florencia Varela, Kristin Lampe, Kendall P. Murphy, Chia-Ying Lin, Jose L. Peiro

**Affiliations:** ^1^Center for Fetal and Placental Research, Cincinnati Children’s Hospital Medical Center (CCHMC), Cincinnati, OH, United States; ^2^Department of Surgery, College of Medicine, University of Cincinnati, Cincinnati, OH, United States; ^3^Department of Orthopaedic Surgery, College of Medicine, University of Cincinnati, Cincinnati, OH, United States

**Keywords:** neural tube defect (NTD), spina bifida (SB), neural progenitor cell (NPC), astrogenesis, Pax6, Olig2, Nkx2.2

## Abstract

During embryonic spinal cord development, neural progenitor cells (NPCs) generate three major cell lines: neurons, oligodendrocytes, and astrocytes at precise times and locations within the spinal cord. Recent studies demonstrate early astrogenesis in animal models of spina bifida, which may play a role in neuronal dysfunction associated with this condition. However, to date, the pathophysiological mechanisms related to this early astrocytic response in spina bifida are poorly understood. This study aimed to characterize the development of early astrogliosis over time from Pax6+, Olig2+, or Nkx2.2+ NPCs using a retinoic acid-induced spina bifida rat model. At three gestational ages (E15, E17, and E20), spinal cords from fetuses with retinoic acid-induced spina bifida, their healthy sibling controls, or fetuses treated with the vehicle control were analyzed. Results indicated that premature astrogliosis and astrocytic activation were associated with an altered presence of Pax6+, Olig2+, and Nkx2.2+ NPCs in the lesion compared to the controls. Finally, this response correlated with an elevation in genes involved in the Notch-BMP signaling pathway. Taken together, changes in NPC patterning factor expression with Notch-BMP signaling upregulation may be responsible for the altered astrogenesis patterns observed in the spinal cord in a retinoic acid-induced spina bifida model.

## Introduction

Spina bifida, characterized by the failure of the neural tube to close fully during embryonic development, is a defect that leads to neurological and physiological disabilities in the fetus, including motor, urinary, intestinal, and sexual dysfunctions, as well as impaired mental development (Mitchell et al., 2004). Often referred as the “two-hit” hypothesis, the primary neural tube defect is followed by *in utero* neurodegeneration secondary to mechanical and chemical trauma (Meuli and Moehrlen, 2014). The components of the amniotic fluid, such as meconium, likely contribute to this neurodegeneration with advancing gestation, which is supported by the significant neurologic improvement observed after *in utero* repair of the defect (Adzick et al., 2011; Moldenhauer and Adzick, 2017). A better understanding of this neural injury’s progression will aid in the development of targeted therapeutics that can combat neurodegeneration.

During normal spinal cord development in the fetus, the ventricular zone (VZ) contains multipotent neural progenitor cells (NPCs) that generate three major cell lineages: neurons, oligodendrocytes, and astrocytes. Moreover, the fate of each cell type depends on the time in which NPCs are initiated to differentiate and their location in the neuroepithelial (Temple, 2001). In normal conditions, neurogenesis occurs in the early embryonic stage, and astrogenesis and oligodendrogenesis occur later in development and even after birth (Reemst et al., 2016). However, recent studies demonstrate that astrocyte differentiation, proliferation, and activation occur earlier in the spinal cord of animals with chemically induced spina bifida (Oria et al., 2018). This early astrocytic reaction may play a role in the impaired neurogenesis and, therefore, the decline in overall neural tissue function. To date, the pathophysiological mechanisms related to this early astrocytic response in spina bifida are poorly understood. However, we anticipate that the regulation of NPC differentiation plays a critical role in this reaction.

Complex interactions between intracellular transcriptional regulators and extracellular signals define the timing and the location of NPC differentiation. For example, transcription factors in the ID and Hes gene families have been implicated as master regulators of normal astrocyte differentiation (Bertrand et al., 2002). Additionally, homeodomain factors, including Pax6 and Nkx2.2, and basic helix loop helix factor, Olig2, determine the differentiation fate of NPCs located on the dorsoventral wall. Moreover, NPCs located on the dorsoventral wall also differentiate depending on bone morphogenetic proteins (BMPs), produced by the roof plate, and sonic hedgehog (Shh), produced by the floor plate (Ulloa and Briscoe, 2007; Molofsky et al., 2012). These morphogens are key regulators of NPC differentiation potential toward neuron and glia cell lineages (Dennis et al., 2019). Interestingly, changes in these transcriptional and extracellular signals may be a possible mechanism for earlier astrocyte presence in spina bifida; however, this has not yet been investigated.

This study characterizes the expression of transcription factors and extracellular signals that regulate NPC differentiation at three time points during gestation (E15, E17, and E20) in a retinoic acid (RA)-induced rat model of *in utero* spina bifida. Importantly, we demonstrate a possible implication of Pax6, Nkx2.2, and Olig2 transcription factors, as well as factors in the Notch-BMP signaling pathway, in the premature shift of NPCs into astrocytes in fetuses with RA-induced spina bifida. These results may lead to new possible therapeutic targets for regulating neurogenesis and gliogenesis in patients with spina bifida.

## Materials and Methods

The experimental protocols were in agreement with the National Institutes of Health Guidelines for Care and Use of Laboratory Animals and were approved by the Institutional Animal Care and Use Committee at Cincinnati Children’s Hospital Medical Center (IACUC 2019-0081).

### Congenital Retinoic Acid-Induced Spina Bifida Animal Model and Experimental Design

The study was performed using 36 timed-pregnant Sprague-Dawley rats weighing 200–250 g (Charles River Laboratories, Inc., Wilmington, MA, United States) and housed at 22°C in a standard dark:light cycle (10:14 h) (light 7:00–19:00) with access to water and standard food *ad libitum*. Mating date was defined as E1 and plug day as E0. Trans-retinoic acid (RA) (Sigma-Aldrich Chemical, St. Louis, MO, United States) was solubilized in olive oil (vehicle) at room temperature, protected from light, and used within 1 h of preparation. Twenty rats were gavaged with RA (100 mg/kg) and 16 rats with an equal volume of the vehicle on E10 at 10 a.m. With this model, 70% of fetuses in each liter were diagnosed with spina bifida (Oria et al., 2018).

The following groups were assessed at three gestational ages (E15, E17, and E20):

1. MMC: open spinal cords from fetuses with RA-induced spina bifida (*n* = 6–8 per time point).
2. Control: spinal cords from non-affected siblings of RA-treated rats (*n* = 6–8 per time point).
3. Vehicle: spinal cords from fetuses whose mothers received vehicle (olive oil) (*n* = 6–8 per time point).

### Tissue Processing

At each gestational time point (E15, E17, and E20), spinal cords from vehicle, control, and MMC fetuses were dissected, snap-frozen, and stored at −80°C until analyzed for gene expression. For histological analysis, spinal cords were dissected and fixed in 4% paraformaldehyde for 24 h and processed for paraffin embedding.

### RNA Extraction and RT-qPCR Analysis

Frozen spinal cords were suspended in RLT buffer and then homogenized using an IkaT10 basic Ultra-Turrax homogenizer. RNA was extracted using the RNeasy Plus Mini Kit (Qiagen Science, Hilden, Germany) following manufacturer’s protocol and RNA quantity was assessed through spectrophotometric analysis using an Epoch Biotek spectrophotometer (Biotek Instruments, Winooski, VT, United States). Utilizing the RT^2^ First Strand Kit (Qiagen Sciences, MD, United States), 1 μg RNA/sample was reverse transcribed into cDNA. A 1-μg cDNA sample was then used as a template for RT-qPCR employing TaqMan^®^ gene expression assays (Applied Biosystems, Foster City, CA, United States) (Supplementary Table 1) in the 7500 Fast Real-Time PCR System. Samples were ran in duplicate for target genes and were normalized using HPRT1 as an endogenous control. Relative quantification of transcript expression was performed using the 2^–ΔΔCt^ method where C_*t*_ represents the threshold cycle.

### RNA-seq Data Processing and Analysis

Raw FastQ files from the RNA-seq data Sequence Read Archive (SRA)^1^ and BioProject (PRJNA683230) and SRA (PRJNA683793) published by Murphy et al. (2021) were processed through the AltAnalyze package v2.0^2^ (Emig et al., 2010). The GOseqR package was used to perform gene ontology (GO) enrichment analysis and GO terms with a corrected *p*-value less than 0.05 were considered statistically significant. This analysis identified differentially expressed genes between control, vehicle, and MMC groups at E15, E17, and E20 (Emig et al., 2010). The analysis was conducted according to the functional annotation in “Neurogenesis” genes in the GO database (GO:0022008). Additionally, analysis was conducted according to the functional annotation “Astrocyte Differentiation” genes in the GO database (GO:0048708) and “Oligodendrocyte Differentiation” in the GO database (GO:0048709), sub-classifications within the “Neurogenesis” GO database.

### Immunostaining

Sections were deparaffinized, rehydrated, and incubated in sodium citrate buffer (pH 6) for 30 min at 95°C to retrieve antigens. Sections were permeabilized with 0.5% Triton X-100 (Sigma-Aldrich, St. Louis, MO, United States) in phosphate-buffered saline (PBS) and incubated in 3% peroxide for 15 min at room temperature. Non-specific binding was blocked for 1 h with 5% BSA in PBS at room temperature, and sections were then incubated overnight at 4°C in a humidity chamber with the following primary antibodies: anti-Olig2 (Millipore, #AB9610 Rabbit and #ABE1024, Guinea Pig) (1:1,000), anti-GFAP (Abcam, #AB4674, Chicken) (1:500), anti-Pax6 (Abcam #ab5790, Rabbit) (1:1,000), anti-Nkx2.2 (Novus Biological, #NBP2-34799, Mouse) (1:500), anti-Nestin (BD Biosciences, #556309, Rat) (1:50), anti-Vimentin (Sigma, #V6630, Mouse) (1:200), BMP-4 (Invitrogen, #PA5-19683, Rabbit) (1:800), BMP-2 (Invitrogen, #PA5-78874, Rabbit) (1:250), S100b (Sigma, #SAB4200671, Mouse) (1:500), Tubulin β III (TUBB3) (Abcam, #ab18207, Rabbit) (1:500) Aldh1l1 (Novus Biological #NBP2-50045, Mouse) (1:1,000), AQP4 (Sigma #A4971, Rabbit) and Doublecortin (DCX) (Invitrogen #PA5-17428, Rabbit) (1:200). Sections were washed and incubated for 1 h with Alexa Fluor 488 (#1531671, Donkey and #1531669 Goat, #1990462, Goat), Alexa Fluor 568 (#1691230, Goat, #1398018, Goat, #1504529, Goat), or Alexa Fluor 647 (#1445259, Goat, #1608641, Donkey) conjugated secondary antibodies (Life Technologies) (1:1,000) in the dark at room temperature in a humidity chamber. Slides were washed, covered with mounting media containing DAPI (Southern Biotech, Birmingham, AL, United States), and visualized with a Nikon fluorescent microscope (Nikon Inc., Melville, NY, United States).

### Immunolabeled Cell and Area Quantification

Pax6+, Olig2+, Nkx2.2+ cell counts, and GFAP+ immuno-stained area measurement was done using NIS Elements AR 4.5 software (Nikon Instruments Inc.). Quantification was conducted using more than ten random high magnification images per spinal cord section in three consecutives sections from each animal. This analysis was conducted in 4–6 animals per group. Cell count data is reported as the percentage of immuno-positive cells compared to the total number of cells in each area. Area is reported as the positively stained area in square pixels as a percentage of the total area. All quantifications were performed by an investigator blinded to the experimental groups.

### Statistical Analysis

All statistical analysis and graphs were performed in Graph Pad Prism 9 software (GraphPad Software Inc., La Jolla, CA, United States). Differences among multiple groups were analyzed by one-way analysis of variances (ANOVA) using Turkey’s *post hoc* test. Differences among the same group at different time points were analyzed by two-way ANOVA using Tukey’s *post hoc* test. Results are reported as means ± standard error (SE) for the relative gene expression (2^–ΔΔCt^) and means ± standard deviation (SD) for all cell counting analysis. A *p*-value < 0.05 was considered statistically significant.

## Results

### Precautious Astrocyte Generation in Spina Bifida

Altered cell quantification of astrocytes (GFAP) and neurons (NeuN) expression has been described in spina bifida *in utero* (Reis et al., 2007; Danzer et al., 2011; Oria et al., 2018), but the correlation with the NPC which are the origin of these differentiated cell types it is not understood in spina bifida physiopathology. In previous mentioned works neuronal numbers decreased during gestation in spina bifida. To determine the neuronal fate and the distribution of premature neurons, we stained spinal cords with Class III β-tubulin and Doublecortin (Dcx) markers of progenitor cells committed to a neuronal fate. In rat fetuses, spina bifida led to extensive loss of Class III β-tubulin and Dcx as gestation progresses suggesting decrease in the expression of premature neurons compared to the controls with normal development (Supplementary Figure 1).

**FIGURE 1:**
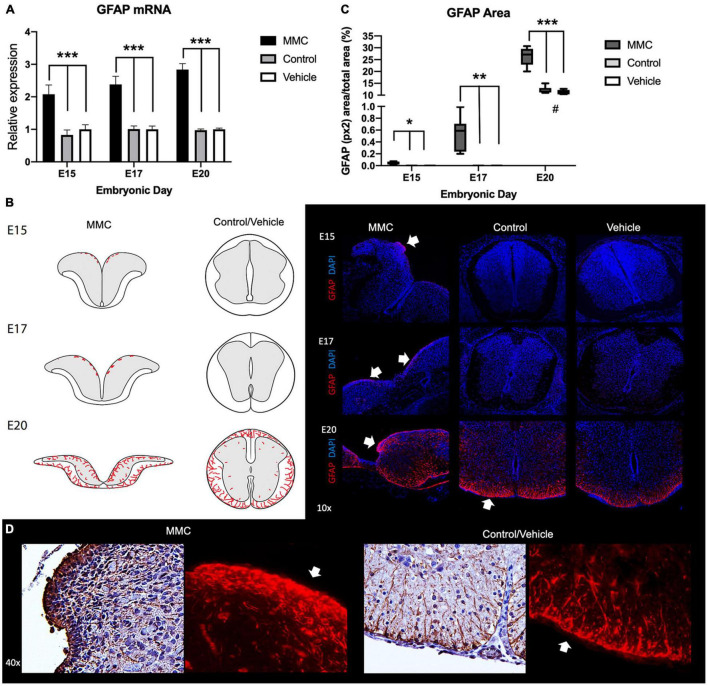
Progressive immunoreactive astrocytes in spina bifida fetuses. **(A)** GFAP relative expression increased in spina bifida (MMC) compared with control and vehicle spinal cords. Values (means ± SE, 6 fetuses/group) of relative expression (2^– ΔΔCt^) for RA-treated and time-matched controls (***p < 0.001). **(B)** Schematic representation of GFAP distribution in MMC and control fetal spinal cords at E15, E17, and E20 (left). GFAP staining (right) in MMC, control, and vehicle fetal spinal cords at E15, E17, and E20. Progressive GFAP+ (red) DAPI+ (blue) immunoreactive astrocyte cells at E15, E17, and E20 in exposed spina bifida (arrows) (10× images). **(C)** GFAP stained area/total picture area in all groups at three gestational ages E15, E17, and E20 (means ± SD, *p < 0.05, **p < 0.01, ***p < 0.001, ^#^*p* < 0.05 between control and vehicle groups at E20 compared to E15 and E17). **(D)** GFAP-immunoreactive astrocytes in spina bifida (MMC) VZ exposed to the amniotic fluid compared to filamentous astrocyte projections in white matter control spinal cords at E20 (40X).

Motivated by evidence that neural cell fate differentiation processes are impacted, with decreased neurogenesis, decreased oligodendrogenesis, and increased astrogenesis, as the RNA-seq (Murphy et al., 2021) and previous work in spina bifida in rats (Oria et al.,. 2018) are described; we investigated how astrocyte presence in the fetal spinal cord at various time points was affected by exposure of the NPC to the amniotic fluid in spina bifida. Not only has GFAP been used in immunohistochemical studies to characterize changes in reactive astrocytosis, but it has also been used in developmental studies as a major mature-astrocyte marker (Koyama, 2014) and therefore was our primary indicator of astrocytes. Interestingly, we observed significant robust upregulation of astrocyte marker, glial fibrillary acidic protein (GFAP), gene expression in MMC fetuses compared to vehicle and control at each studied time point (^***^p < 0.001) (Figure 1A). Similarly observed in the RNA-seq results with upregulated GFAP expression at all three time points (E15, E17, and E20) but not significant at E17 and E20 (Supplementary Table 2). Immunofluorescent staining indicates that this upregulation correlates with an elevation in the area stained with GFAP in MMC at E15 and E17 before any GFAP-positive cells are detected in normal control groups suggesting the accelerated gliogenesis altered process by spina bifida immature spinal cord (*p < 0.05, ^**^p < 0.01, ^***^p < 0.001) (Figures 1B,C). As observed in normal development (Yoon et al., 2017) after E18.5 but mostly at E21 and postnatal, astrocytes beneath the pial surface begin expressing GFAP, which is evident in control and vehicle fetuses where more area is positive for GFAP at E20 compared to E15 and E17 (^#^*p* < 0.05, control and vehicle E20 vs. E15 and E17) (Figures 1B,C). Importantly, early expression of GFAP is observed in the open spinal cords of MMC fetuses that are exposed to the amniotic fluid (Figure 1B). This area corresponds to the VZ and sub-ventricular zone (SVZ), where NPCs are normally located and responsible for neural development processes. In this VZ, we observed distribution of GFAP-hypertrophic positive astrocytes appeared as numerous aggregates throughout the lesion near the exposed neural tissue in spina bifida fetuses. In addition, glial cells (GFAP) do not express in the VZ of control group and the expression in the white matter are not hypertrophic and showed elongated bodies and long filamentous projection (Figures 1B,D).

**FIGURE 2:**
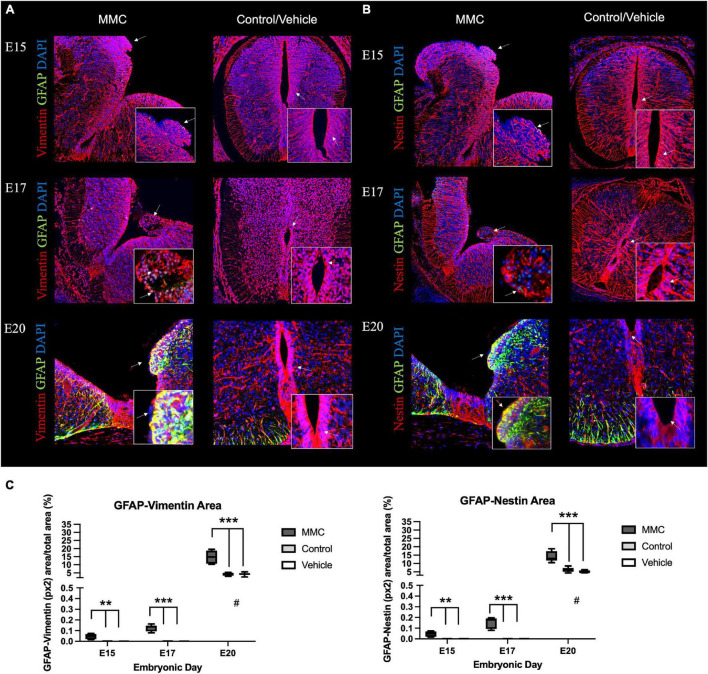
*In utero* progressive astrogliosis in spina bifida. Progressive early astrogliosis in spina bifida (MMC) at E15, E17, and E20 compared with control spinal cords. Progressive GFAP (green) immunoreactive astrocyte cells, **(A)** vimentin (red) and **(B)** nestin (red) reactive astrocytes co-localization (yellow) and DAPI (blue) at E15, E17, and E20 in spina bifida (arrows) (20×). Control/Vehicle immunoreactive GFAP (green) astrocytes in white matter beneath the pial surface at E20 in normal spinal cords (20×). Astrocytes GFAP (green) co-localized with **(A)** vimentin (red) and **(B)** nestin (red) in spina bifida fetuses at in the VZ region compared with controls/vehicle with no expression of GFAP in the VZ nor central canal (arrow). **(C)** GFAP + Nestin and GFAP + Vimentin-stained area/total picture area in all groups at three gestational ages E15, E17, and E20 (means ± SD, **p < 0.01, ***p < 0.001, ^#^*p* < 0.05 between control and vehicle groups at E20 compared to E15 and E17).

Next, we investigated the reactivity of this GFAP+ astrocyte population in spinal cord sections from MMC and control groups, as reactivity is a major feature of spinal cord injury. This reactivity was determined based on co-expression of GFAP, vimentin, and nestin after immunostaining. Under normal spinal cord development, early neuroepithelial progenitors in the spinal cord and radial glial cells express nestin and vimentin, as we observed in vehicle control and RA control spinal cord tissue sections at E15 (Figure 2). Numerous processes in the dorsal and ventral halves radiating from the central canal, corresponding to radial glial, express vimentin, and nestin (Barry and McDermott, 2005) (Figures 2A,B). Although you only see that during embryonic development (radial glial) and then very rarely in adult neurogenic zones. At E20 in healthy fetal spinal cords, GFAP is highly expressed in the white matter; however, GFAP+ cells (green) are deemed unreactive as GFAP staining does not co-localize completely with nestin and vimentin. In these representative sections, nestin and vimentin are predominantly expressed in the white matter’s radial astrocytes and attenuated radial glial cells of the gray matter. In contrast, we observed hypertrophic and reactive astroglia in MMC fetal spinal cords as indicated by GFAP co-localization with vimentin and nestin (yellow) as early as E15 but is a prominent response by E20 (Figure 2B, white arrows). GFAP co-localization with nestin and vimentin was observed in focal points at E15 and E17 when GFAP expression was not detected in control/vehicle tissues.

In addition, this reactive astroglia was predominantly found in the VZ as well as the medial septum and radial processes, areas directly exposed to the amniotic fluid as a result of the defect and different expression pattern was evident compared to control groups (Figures 2A,B, white arrows). Furthermore, immunofluorescent staining indicates an increase in the area stained with GFAP + Nestin and GFAP + Vimentin in MMC fetuses at the different time points even before any GFAP-positive cells are detected in normal control groups (E15 and E17) suggesting the accelerated gliogenesis and activation process by spina bifida immature spinal cord (^**^p < 0.01, ^***^p < 0.001) (Figure 2C). In normal development at E21 astrocytes beneath the pial surface in the white matter begin expressing GFAP, also some area is positive for GFAP + Nestin or GFAP + Vimentin compared control but also the distribution is not the same location as in MMC is located in the VZ. This increase in co-expression of GFAP and Nestin or Vimentin also is increased at the end of gestation compared to E15 and E17 (^#^*p* < 0.05, control and vehicle E20 vs. E15 and E17) (Figure 2C).

**FIGURE 3:**
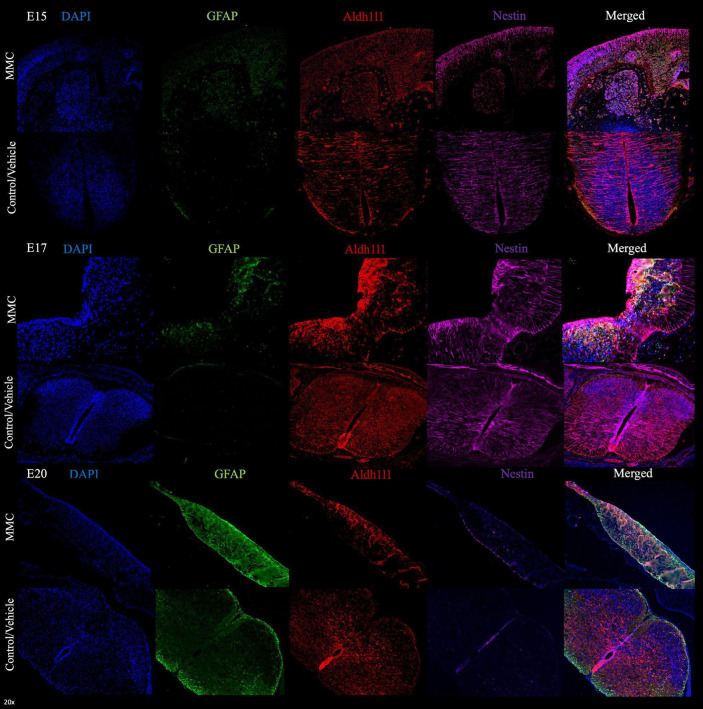
Premature astrocytes in spina bifida. Progressive early astrogliosis in spina bifida (MMC) at E15, E17, and E20 compared with control spinal cords. GFAP (green), Aldh1l1 (red), Nestin (magenta), and DAPI (blue) premature astrocytes localization in VZ exposed to amniotic fluid at E15, E17, and E20 in spina bifida (20×).

**FIGURE 4:**
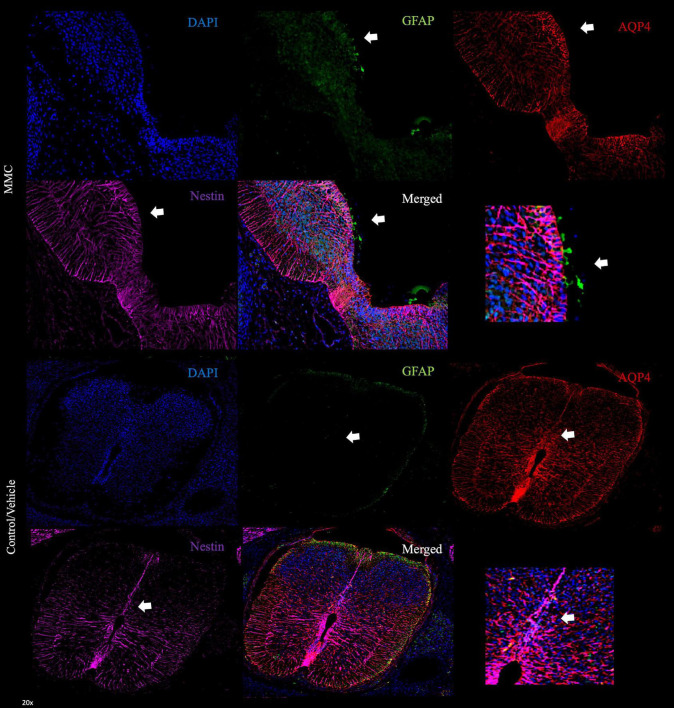
Aquaporin 4 astrocytes in ventricular zone spina bifida fetuses. Generation of mature astrocytes in MMC compared with control spinal cords at E17. AQP4+ GFAP+ cells located in the VZ exposed to the amniotic fluid in spina bifida at E17 (20×). Magnification of VZ in MMC and control/vehicle animals (arrow).

To further validate the expression of astrocytic markers and astroglia reactivity in the VZ exposed to the amniotic fluid in MMC fetuses were determined using immunostaining with other premature astrocyte markers as Aldh1l1 and Aquaporin 4 (AQP4). Using both markers we observed and increase of expression at E17 gestational age (Figures 3, 4). The expression of AQP4 and Aldh1l1 decreased at E20 which correlated with the increase of GFAP expression, used as more mature astrocyte marker (Figures 3, 4). The expression of both Aldh1l1 and AQP4 were observed in the VZ, niche of the NPC and exposed to the amniotic fluid in MMC fetuses.

### Gene Ontology Functional Analysis of Differential Expressed Genes in Neurogenesis

**FIGURE 5:**
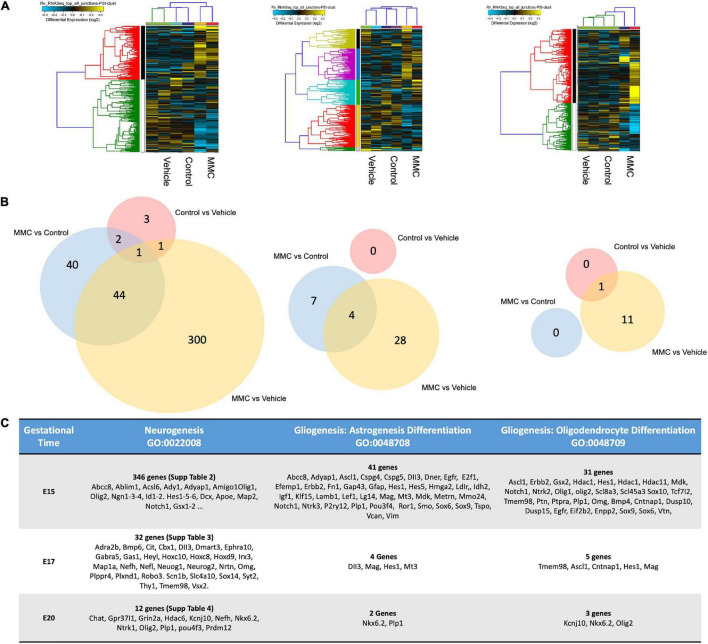
Cluster analysis of differentially expressed genes. **(A)** Hierarchical cluster analysis of differentially expressed genes showed transcriptome-wide expression patterns similar between the control and vehicle groups compared to MMC samples in all three gestational ages E15, E17, and E20 days. **(B)** Venn diagram of the DEGs in different comparisons. The numbers indicate unique and common DEGs in two duplicates for different comparisons MMC vs. VC, MMC vs. NC and NC vs. VC. **(C)** Analysis of two sub-classifications within the Neurogenesis GO, Oligodendrocyte Differentiation (GO:0048709), and Astrocyte Differentiation (GO:0048708) DEGs between the control and vehicle groups compared to MMC at E15, E17, and E20 days.

To gain molecular insights into the injury responses that are mediated by the exposure of the neural tissue to the amniotic fluid in spina bifida *in utero*, we conducted transcriptome analysis of existing RNA-seq datasets from lumbar spinal cords of fetuses in each experimental group at E15, E17, and E20 (Murphy et al., 2021). Gene expression levels were compared between fetuses with MMC and their control siblings (control) or those that received olive oil (vehicle). Hierarchical cluster analysis of differentially expressed genes demonstrated transcriptome-wide expression patterns that were similar between spinal cords collected from vehicle and control fetuses compared to those from MMC at all three gestational ages: E15, E17, and E20 days (Figure 5A) as published by Murphy et al. (2021). An analysis from the RNA-seq study was conducted according to the functional annotation in “Neurogenesis” genes in the GO database (GO:0022008). From the 4,975 annotations, we identified 388 genes that were expressed differently between MMC and the two control groups at E15 (Supplementary Table 3 and Figure 5B), 39 genes were expressed differently between MMC and the two control groups at E17 (Supplementary Table 4 and Figure 5B), and 12 genes were expressed differently between MMC and the two control groups at E20 (Supplementary Table 5 and Figure 5B). Finally, only seven genes were differentially expressed in the neurogenesis GO between vehicle and control at E15 (Supplementary Table 3 and Figure 5B), zero at E17 (Figure 5B), and one at E20 (Supplementary Table 5 and Figure 5B).

Analysis of two sub-classifications within the Neurogenesis GO, Oligodendrocyte Differentiation (GO:0048709), and Astrocyte Differentiation (GO:0048708), was conducted to elucidate the early changes in gene expression that could change the NPC fate. Within Oligodendrocyte Differentiation (GO:0048709), we identified 31 genes differentially expressed between MMC and control groups at E15, 5 at E17 and 3 at E20 from the 174 annotations. At early stages, E15, we identified changes in genes in oligodendrocyte differentiation (Olig1, Olig2, Slc8a3, Sox10, Tmem98, Bmp4, Dusp10, Dusp15, Eif2b2, Enpp2, Errbb2, Gsx2, Hdac1, Hes1, Il34, Mag, Mdk, Med12, Nkx6-1, Notch1, and Ntrkn1), oligodendrocyte development (Tcf7l2 and Ascl1), myelinization (Cntnap1) when compared to controls (Figure 1C). Additionally, within Astrocyte Differentiation (GO:0048708), we identified 41 genes dysregulated in MMC vs. control groups at E15, 4 genes at E17 and 2 genes at E20 (Figure 5C) from the 424 annotations. Examining the differentially expressed genes in the Astrocyte Differentiation GO, we observed early changes at E15 in genes involved in the regulation of glial cell proliferation (Gfap, Notch1, Vim, Abcc8, Adyap1, and E2f1), glial cells differentiation (Ascl1, Dner, Erbb2, Gap43, Hes1, Igf1, Klf15, Lef1, Metrn, Mmp24, Plp1, and Bmp4), glial cell migration (Cspg4, Efemp1, Fn1, Idh2, lamb1, P2ry12, Tspo, and Vcan) glial cell projection (Cspg5 and Mdk), glial cell fate commitment (Hes5), glial cell development (Lgi4), astrocyte differentiation (Dll3, Hes1, Hes5, Hmga2, Mag, Notch1, Ntrk3, and Sox6), astrocyte activation (Egfr, Ldrl, and Smo), astrocyte development (Mt3, Plp1, Pou3f2, Ror1, and Vim), and astrocyte commitment (Sox9) compared to controls (Figure 5C). Validation of the RNA-seq data was performed using RT-qPCR for some of the key genes in astrogenesis (Supplementary Table 2).

### Altered Patterning Factors in the Ventricular Zone in Spina Bifida

**FIGURE 6:**
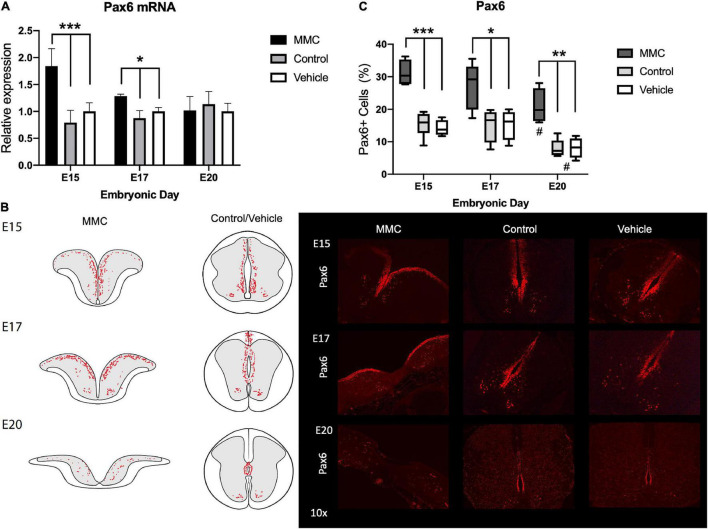
Pax6 expression and distribution in spina bifida. **(A)** Pax6 relative expression increased in spina bifida (MMC) compared with control and vehicle spinal cords at E15 and E17. Values (means ± SE, 6 fetuses/group) of relative expression (2^– ΔΔCt^) for RA-treated and time-matched controls (*p < 0.05, ***p < 0.001). **(B)** Schematic representation of Pax6 distribution in MMC and control fetal spinal cords at E15, E17, and E20 (left). Pax6+ staining in MMC, control, and vehicle spinal cords at E15, E17, and E20 (right) (10×). Pax6+ (red) immunoreactive NPC cells exposed at E15, E17, and E20 in spina bifida (10×). **(C)** % Pax6+ NPC in spina bifida, control and vehicle spinal cords determined by Pax6 stained cells/total cells at three gestational ages E15, E17, and E20 (means ± SD, *p < 0.05, **p < 0.01, *p < 0.001, ^#^*p* < 0.05 between E20 and E15).

In the developing spinal cord, the VZ is divided into three domains along the dorsoventral axis: Pax6 (p0-2), Olig2 (pMN), and Nkx2.2 (p3) domains (Shirasaki and Pfaff, 2002). During spinal cord development, these patterning domains are established early in gestation, by E11, and their expression remains during the course of neuro- and gliogenesis until astrogenesis begins in the end stages of gestation (Sugimori et al., 2007). We hypothesize that treating rats with RA at E10 will modulate the spinal cord’s patterning domains. Therefore, to assess the impact of RA-induced spina bifida on NPC differentiation, we characterized Pax6, Olig2, and Nkx2.2 expression in fetal spinal cords during gestation because in spina bifida the spinal cord is open and unfolded exposing the VZ and the progenitor cells to the amniotic fluid. First, Pax6 gene expression was upregulated in MMC at E15 and E17 compared to control and vehicle groups but returns to normal levels by E20 (*p < 0.05, ^**^p < 0.01) (Figure 6A). At E15 and E17, Pax6+ NPCs are located in the VZ in all groups; however, those found in the MMC group are directly in contact with the amniotic fluid as a result of the defect. At E20, Pax6 expression is restricted to the ependyma located in the central canal in fetuses from both control and vehicle groups; in contrast, Pax6+ cells in MMC fetuses are spread throughout the gray matter in both halves of the spinal cord (Figure 6B). Upon quantification of immunofluorescence staining, we observed a significant increase in Pax6+ cells in MMC fetal spinal cords compared to the control groups at each time point (*p < 0.05, ^**^p < 0.01, ^**^p < 0.001) (Figure 6C). Interestingly, by E20, the percentage of Pax6+ cells decreased in control and vehicle compared to E15 (^#^*p* < 0.05) following the normal decrease in expression during spinal cord development associated with oligodendrogenesis and astrogenesis (Sugimori et al., 2007) (Figure 6C). The percentage of Pax6+ cells in the MMC group also decreased during gestation (^#^*p* < 0.05 E15 vs. E20), however, it still was significantly higher compared to the other groups (Figure 6C).

**FIGURE 7:**
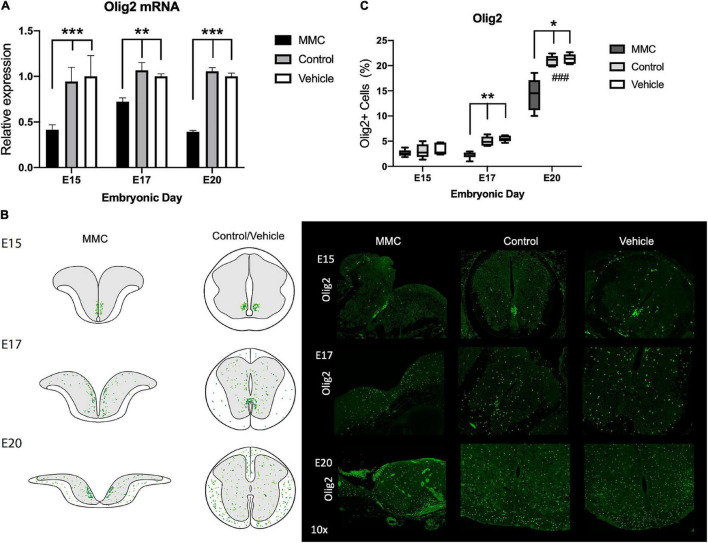
Olig2 expression and distribution in spina bifida. **(A)** Olig2 relative expression increased in spina bifida (MMC) compared with control and vehicle spinal cords at E15 and E17. Values (means ± SE, 6 fetuses/group) of relative expression (2^– ΔΔCt^) for RA-treated and time-matched controls (*p < 0.05, **p < 0.01, ***p < 0.001). **(B)** Schematic representation of Olig2 distribution in MMC and control spinal cords at E15, E17, and E20 (left). Olig2+ staining in MMC, control, and vehicle spinal cords at E15, E17, and E20 (right) (10×). Olig2 (green) immunoreactive NPC cells exposed at E15, E17, and clustering at E20 in spina bifida (10×). **(C)** % Olig2+ NPC in spina bifida (MMC), control, and vehicle fetal spinal cords determined by Olig2 stained cells/total cells at three gestational ages E15, E17, and E20 (means ± SD, *p < 0.05, **p < 0.01, ^###^*p* < 0.001 between control and vehicle groups at E20 compared to E15 and E17).

Down-regulation of Olig2 patterning factor was observed in MMC fetuses compared with control and vehicle animals at each time point studied (^**^p < 0.01, ^***^p < 0.001) (Figure 7A), similarly observed in the RNA-seq results (Supplementary Tables 3–5). During spinal cord development, Olig2+ cells migrate from the pMN domain to the rest of the spinal cord, as observed in control and vehicle fetuses at E15, E17, and E20 (^###^*p* < 0.001, E20 vs. E15 and E17) (Figures 7B,C). In contrast, with MMC fetuses, Olig2+ cells remained as tight clusters in the VZ over this gestational period and were exposed to the amniotic fluid (Figure 7B). Furthermore, down-regulation of Olig2 mRNA and clustering of Olig2+ cells within the VZ observed in MMC is associated with a decrease in the number of Olig2+ cells at E17 and E20 as compared to control and vehicle (*p < 0.05) (E15, E17, and E20) (Figure 7C). There was no difference in the percentage of Olig2+ cells between groups at mid gestation (E15). Interestingly, the Olig2 (pMN) domain was exposed to the amniotic fluid in most samples which may lead to this response.

**FIGURE 8:**
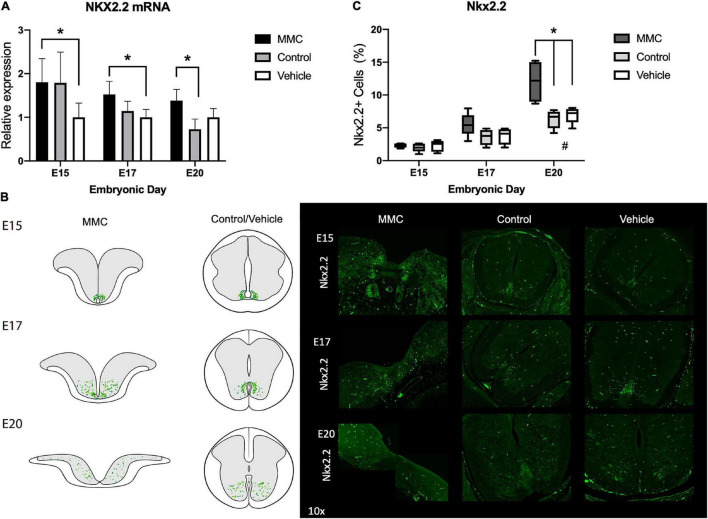
Nkx2.2 expression and distribution in spina bifida. **(A)** Nkx2.2 relative expression increased in spina bifida compared with control and vehicle spinal cords at E15 and E17. Values (means ± SE, 6 fetuses/group) of relative expression (2^– ΔΔCt^) for RA-treated and time-matched controls (*p < 0.05). **(B)** Schematic representation of Nkx2.2 distribution in MMC and control spinal cords at E15, E17, and E20 (left). Nkx2.2+ staining in MMC, control, and vehicle spinal cords at E15, E17, and E20 (right) (10×). Nkx2.2 (green) immunoreactive NPC cells at E15, E17, and E20 in spina bifida (10×). **(C)** % Nkx2.2+ NPC in MMC, control, and vehicle spinal cords determined by Nkx2.2 stained cells/total cells at three gestational ages E15, E17, and E20 (means ± SD, *p < 0.005, ^#^*p* < 0.05 between control and vehicle groups at E20 compared to E15 and E17).

In spina bifida fetuses Nkx2.2 transcription factor was upregulated at E15 and E17 when compared to vehicle spinal cords and upregulated at E20 when compared to control (*p < 0.05) (Figure 8A). In normal development, Nkx2.2 cells are restricted to the p3 domain of the ventromedial column in early gestational ages. As our data supports, during normal development, NPCs begin to migrate to the ventral spinal cord as early as E13 and by E15, Nkx2.2 cells can be detected in the ventral gray matter (to differentiate into ventral interneurons) and in the white matter (to differentiate into oligodendrocytes). By the end of gestation, Nkx2.2 cells can be detected mostly in the ventral region (McMahon, 2000; Qi et al., 2001; Figure 8B). Even with the defect early in gestation, Nkx2.2. cells remain restricted to the p3 domain at E15 in the MMC group; however, they begin to migrate at E17, and are ultimately found throughout the entire spinal cord by E20 (Figure 8B). In addition, upon quantification of immunofluorescence staining, we observed a significant increase in Nkx2.2+ cells during development (^#^*p* < 0.05) (Figure 8C, E20 vs. E15 and E17), and an increase in MMC compared to both control groups at E20 (*p < 0.05) (Figure 8C). As shown in previous studies (Mizuguchi et al., 2001; Sugimori et al., 2007), the down-regulation of Olig2 resulted in up-regulation of Nkx2.2 and dorsal expansion of Nkx2.2 + domain. Consequently, more Nkx2.2 cells and fewer Olig2+ cells were detected at E17 and E20 in the spinal cord of MMC fetuses compared with Control and Vehicle spinal cords (*p < 0.05) (E15, E17, and E20) (Figures 8B,C).

### Origin of Premature Astrocyte Population

**FIGURE 9:**
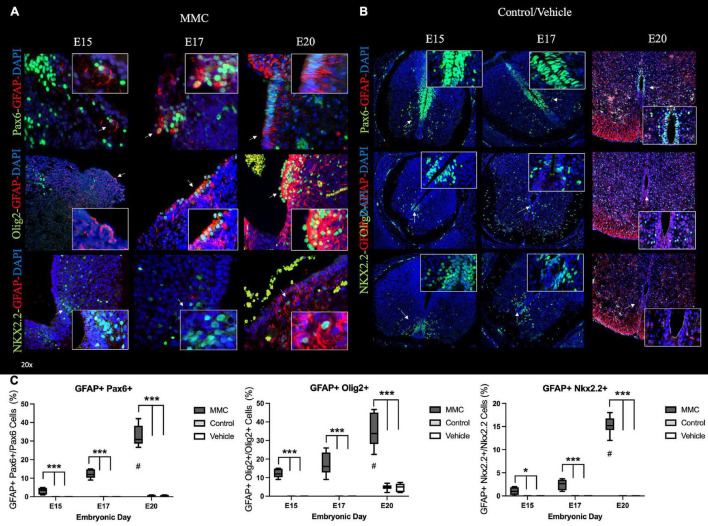
Early NPC differentiation into astrocytes in spina bifida. Progressive early generation of reactive astrocytes in **(A)** MMC compared with **(B)** control spinal cords at E15, E17, and E20. **(A)** Pax6 + GFAP+ cells located in the VZ exposed to the amniotic fluid in spina bifida at E15, E17, and E20 (upper) (arrow). Olig2 + GFAP+ cells located in the VZ exposed to the amniotic fluid in spina bifida at E15, E17, and E20 (center) (arrow). Nkx2.2 + GFAP+ cells located in the VZ exposed to the amniotic fluid in spina bifida at E20 (bellow) (arrow). **(B)** Ventricular zone (VZ) is divided into three domains along the dorsoventral axis: Pax6 (p0-2), Olig2 (pMN), and Nkx2.2 (p3) domains and GFAP sub-pial expression in normal spinal cords. Pax6 (green), Olig2 (green), Nkx2.2 (green), GFAP (red), and DAPI (blue) (20× images). **(C)** % GFAP Pax6+, GFAP+ Olig2+ and % GFAP+ Nkx2.2 astrocytes in spina bifida, control and vehicle spinal cords determined by GFAP and Pax6, Olig2, or Nkx2.2 stained cells/Pax6, Olig2, or Nkx2.2 at three gestational ages E15, E17, and E20 (means ± SD, *p < 0.05, ***p < 0.001, ^#^*p* < 0.05 between E20 and E15).

Next, we sought to examine the developmental origin of the glial cells in the VZ exposed to amniotic fluid in MMC fetuses by assessing the co-expression of GFAP and patterning factors, Pax6, Olig2, and Nkx2.2, at each time point studied. At E15 and E17, astrocytes (GFAP+ cells) located in the VZ of MMC fetal spinal cords were exposed to amniotic fluid and expressed Olig2 or Pax6 (Figure 9A). At these time points, GFAP expression was not observed in Nkx2.2+ cells around the p0 domain (Figure 9A) (arrows). As previously observed, GFAP expression was not detected in control and vehicle spinal cords at these time points (Figure 9B). At E20, GFAP+ astrocytes found in the VZ of MMC fetal spinal cords expressed Pax6, Olig2, and Nkx2.2 (Figure 9A). On the contrary, GFAP+ cells were found in the white matter at the sub-pial region in control and vehicle spinal cords and did not express Pax6, Olig2, or Nkx2.2 (Figure 9B). This increase in co-expression of GFAP and Pax6, Olig2, and Nkx2.2 compared to controls (*p < 0.05, ^**^p < 0.01, ^***^p < 0.001) (also is increased at the end of gestation compared to E15 and E17 (^#^*p* < 0.05, control and vehicle E20 vs. E15 and E17) (Figure 9C). These results demonstrate the expression of an astrocyte marker (GFAP) in VZ NPC (Pax6, Olig2, and Nkx2.2) that could lead to astrogenesis in the VZ exposed to the amniotic fluid in MMC fetuses.

### Notch Bone Morphogenetic Protein Pathway Involvement in Neural Progenitor Cell Fate in Spina Bifida

**FIGURE 10:**
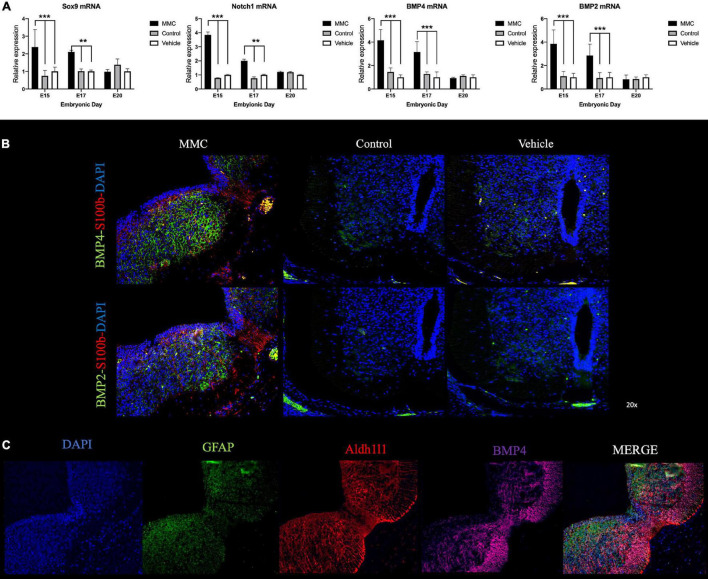
Notch-BMP signaling activation in spina bifida. **(A)** Relative expression of Sox9, Notch1, BMP2, and BMP4 in MMC, control, and vehicle fetuses at E15, E17, and E20. Means ± SE (*n* = 6 fetuses/group) of relative expression (2^– ΔΔCt^) for RA-treated and time-matched controls; (**p < 0.01, ***p < 0.001). **(B)** Co-staining of BMP-2 (green), BMP-4 (green), S100b (red), and DAPI (blue) in spinal cords collected from MMC, control, and vehicle fetuses at E17 (20× images). **(C)** Co-staining of BMP-4 (magenta), Aldh1l1 (red), GFAP (green), and DAPI (blue) in spinal cords collected from MMC at E17 (20× images).

During neural development, Notch plays a crucial role in regulating NPC differentiation into astrocytes by activating different signaling pathways such as Notch, Sox9, and BMP (Fulghum, 2005; Xin et al., 2006). BMP signaling has been shown to be a key player in many events in the central nervous system (CNS) development. For example, BMP2 has been demonstrated to promote astrocytes fate both *in vivo* and *in vitro* (Gross et al., 1996; Bonaguidi et al., 2005). Also, BMP4 has been previously reported to repress oligodendrogenesis and promote astrogenesis during CNS maturation (Gross et al., 1996; Mabie et al., 1999). RNA-seq analysis indicated alterations in BMP signaling earlier in gestation (E15); therefore, in order to determine the involvement of BMP signaling in accelerated astrogenesis in RA-induced spina bifida we assessed the expression of Sox9, Notch1, BMP2, and BMP4 in MMC fetal spinal cords compared to control and vehicle fetal spinal cords. Gene expression of Sox9, Notch1, BMP4, and BMP2 were upregulated in MMC fetal spinal cords compared to control and vehicle groups at E15 and E17 (^**^p < 0.01, ^***^p < 0.001); however, there were no differences between groups at E20 (Figure 10A).

Finally, using immunofluorescence, we investigated if astrocytes were potentially responsible for elevations in BMP-2 and BMP-4 expression. As a marker of early astrocyte maturation, expression of S100b was detected and located in the amniotic fluid exposed layers and the radial glial in MMC samples with almost no expression of S100b in the control groups at this gestational age (E17) (Figure 10B). In corroboration with the gene expression results (Figure 10A), BMP-2 and BMP-4 were more expressed in MMC samples compared to the control groups at the same gestational age (Figure 10B). Moreover, BMP-2 and BMP-4 expressions were primarily located in the gray matter and co-localized with S100b positive cells in the MMC group. In comparison, BMP-2 and BMP-4 expression was diminished and primarily located in the spinal cords ventro-lateral horn in the control groups. Furthermore, observing BMP-4 was high expressed in MMC and using Aldh1l1 as a premature astrocytes marker we observed co-expression of these two markers in MMC fetuses at E17 gestational age (Figure 10C). These results indicate that early S100b and Aldh1l1 positive astrocytes may play a role in BMP-2 and BMP-4 induced premature astrogliosis in MMC fetuses.

## Discussion

In this report, we aimed to elucidate a mechanism behind the premature reactive astrogliosis that occurs in a RA-induced spina bifida rat model with the goal of identifying potential therapeutic targets to repair the prenatal neurodegenerative damage that occurs in the spinal cord after exposure to the intrauterine environment. Our data suggests that neurogenesis changes and reactive astrogliosis occurs earlier in the VZ of spinal cords from fetuses with RA-induced spina bifida compared to control groups.

Astrocyte precursors are generated by differentiation of radial glial cells in early development (Seo et al., 2008) and from migratory progenitors that emerge from the sub-VZ at later in gestation (Levison and Goldman, 1993). Based on our data, we propose that NPCs become committed to the astrocyte lineage due the direct exposure to the amniotic fluid in spina bifida by dysregulation in patterning factors, Pax6, Olig2, and Nkx2.2 and potentially downregulating neurogenesis and oligodendrogenesis. Furthermore, we present the involvement of Notch1, Sox9, and BMP signaling in NPC cell fate during gestation and their role in self-propelling earlier astrogliosis observed in spina bifida. Excitingly, our results suggest that inhibiting NPC differentiation into astrocytes through modulation of Notch/BMP signaling or patterning factors could be a promising neuroprotective strategy.

Astrocytosis has been described previously in other spina bifida animal models (Reis et al., 2007, 2008; Oria et al., 2018); however, the response to injury as early as E15 has not been investigated until now. Therefore, the gestation time points chosen (E15, E17, and E20), when comparing groups studied in this report, provide significant knowledge about the timeline of astrocytosis in spina bifida. Indeed, we demonstrated changes in astrogenesis in spinal cords of fetuses with spina bifida with the presence of differentiated (GFAP) astroglial cells as early as E15 that progressively increase throughout the rest of gestation. This maturation is much earlier than what is observed in healthy conditions, as NPC begins to differentiate into astrocytes around E16.5 and express GFAP, a astroglial marker, by E18.5, which agrees with the timeline of GFAP in our control groups (Ge et al., 2012; Han et al., 2020). These changes were also supported by RNA-seq in which 19 genes were dysregulated at E15, 4 genes at E17, and 2 genes at E20 other than GFAP in the “Neurogenesis” and sub-classification “Astrocyte Differentiation” GO pathway. Additionally, astroglial cells expressed vimentin and nestin in addition to GFAP, AQP4, and Aldh1l1, indicating that the new astrocytes are also reactive (Pekny and Nilsson, 2005; Koyama, 2014; Sofroniew, 2015; Verkhratsky and Parpura, 2016). We propose that exposure to amniotic fluid plays a role in this early and progressive astrogenesis and astrocyte activation affecting neurogenesis and oligodendrogenesis. As a result of failed neural tube closure, a peculiarly variable shaped, uncovered spinal cord is observed, where the dorsal VZ and often the central canal are completely open and directly exposed to the amniotic fluid. Interestingly, differentiated, and reactive astroglial cells were found in the VZ from spina bifida. Astrocytes respond to stress or tissue damage within the CNS by proliferating, activating, and interacting with other cell types through signaling molecules (Alonso, 2005). Therefore, it is possible that these cells are responding to the tissue damage induced by the enzymatic action of the amniotic fluid insult (Botto et al., 1999; Copp et al., 2013; Copp and Greene, 2013; Oria et al., 2019); however, the mechanism behind this reaction remains unclear and should be a focus of future pre-clinical studies.

Next, we focused on understanding how NPC patterning factors and signaling pathways that dictate differentiation fate were altered in spina bifida. NPCs are responsible for normal neurogenesis, oligodendrogenesis, and gliogenesis in the spinal cord during development. Neural cell diversity is a complex process during spinal cord development that is determined by a dorsal and ventral gradient of transcription factors and patterning domains such as Olig2, Pax6, and Nkx2.2 (Jessell, 2000; Rowitch, 2004; Rowitch and Kriegstein, 2010). Our data suggest that this process is altered in fetuses with spina bifida due to modifications in Pax6, Olig2, and Nkx2.2 expression throughout the time points studied. We observed dysregulation in genes involved in the “Astrocyte Differentiation” GO pathway, and the data was validated by measuring specific patterning factors and morphogens by RT-qPCR. Specifically, we observed an upregulation of Pax6 and Nkx2.2 gene expression and an elevated number of Pax6 or Nkx2.2 positive cells in spina bifida fetuses compared to the control groups. In addition, Olig2 patterning domain was downregulated in spina bifida during gestation, and this correlated with fewer Olig2 positive cells. These results relate to previous reports indicating that these modifications lead to enhanced astrogenesis while hindering neurogenesis and oligodendrogenesis in other types of spinal cord injuries. While Pax6 is a proneural patterning factor, its overexpression accelerates neural maturation into early neuronal committed cells but is not associated with net neurogenesis over time. In fact, Pax6 overexpression correlated with a loss of NPCs over time (Klempin et al., 2012). In MMC animals, we demonstrated upregulation of Pax6 in early gestational stages, which correlated with an increase in the number of Pax6+ cells committed to astrocytes, potentially at the cost of neurogenesis. Furthermore, Nkx2.2 has a primary role in ventral neuronal patterning and it has been shown to direct neural cell identity toward the glial lineage (Briscoe et al., 1999; Gorris et al., 2015; Yun et al., 2020). Upregulation of Nkx2.2 has been previously reported in other types of spinal cord injuries (Chang et al., 2009). Olig2 is essential for neurogenesis and oligodendrogenesis; therefore, it is possible that reduction in Olig2 led to enhanced astroglial differentiation as shown previously (Mizuguchi et al., 2001; Zhou et al., 2001; Zhou and Anderson, 2002; Hack et al., 2004; Klempin et al., 2012). In addition to patterning factors, BMP signaling has been shown to regulate astrocyte commitment in the CNS (Furuta et al., 1997; Mehler et al., 1997). In fact, up-regulation of proteins involved in this pathway have been extensively shown to direct NPCs to astroglial fate (Gross et al., 1996; Mabie et al., 1999; Bonaguidi et al., 2005; Miyagi et al., 2012). Our results further support this evidence as Sox9, Notch1, and BMP2/4 gene expression was upregulated at the time of early astrogliosis in spina bifida, and BMP2/4 expression with S100b and Aldh1l1, an early astrocyte markers. Therefore, dysregulation of patterning factors, Olig2, Pax6, and Nkx2.2 in combination with upregulation of Notch/BMP signaling may be a plausible mechanism for the early NPC differentiation into astrocytes as a protective mechanism after injury induced by amniotic fluid exposure.

To date, the neuropathological alterations reported in this congenital RA-induced rat model of spina bifida are (1) presence of early astroglia and progressive astrocytosis (Reis et al., 2007; Danzer et al., 2011; Oria et al., 2018), (2) progressive loss of neurons (Reis et al., 2007; Oria et al., 2018), and (3) neuro-inflammatory response associated with reactive microgliosis (Oria et al., 2018). Future studies are aimed to determine the impact of early astrocytosis on neurons and oligodendrocytes function, as it is hypothesized that NPCs differentiate into astrocytes at the cost of neurogenesis and oligodendrogenesis. If this is the case, developing strategies to prevent premature astrocytosis could improve neural function and overall outcomes in spina bifida patients. Furthermore, our present work identifies several transcriptional targets that would be a promising method to inhibit this astrocytic response.

## Conclusion

In conclusion, spina bifida is one of the most permanently disabling congenital birth defects, and despite fetal surgery to repair or cover the spinal cord at mid-gestation, significant alterations have already been established before the intervention. Identifying the altered mechanism in glio/neurogenesis in response to fetal spinal cord injury in spina bifida is crucial for the development of new therapeutic strategies to improve functional outcomes in these patients. Our results suggest that targeting patterning factors, Pax6, Olig2, and Nkx2.2, as well as BMP/Notch signaling is a promising strategy to impede early gliogenesis in a chemically induced spina bifida rat model.

## Data Availability Statement

The datasets presented in this study can be found in online repositories. The names of the repository/repositories and accession number(s) can be found in the article/Supplementary Material.

## Ethics Statement

The animal study was reviewed and approved by the IACUC 2019-0081.

## Author Contributions

MO and JLP: study concept, design, and supervision. MO, BP, ZL, KB, KG, MV, and KL: acquisition of data. KM, MO, and JLP: analysis and interpretation of data and drafting of the manuscript. KM, MV, MO, C-YL, and JLP: critical revision of the manuscript for important intellectual content. MO: statistical analysis. C-YL and JLP: obtained funding. MO, KM, and KL: technical or material support. All authors contributed to the article and approved the submitted version.

## Conflict of Interest

The authors declare that the research was conducted in the absence of any commercial or financial relationships that could be construed as a potential conflict of interest.

## Publisher’s Note

All claims expressed in this article are solely those of the authors and do not necessarily represent those of their affiliated organizations, or those of the publisher, the editors and the reviewers. Any product that may be evaluated in this article, or claim that may be made by its manufacturer, is not guaranteed or endorsed by the publisher.

## Footnotes

1
https://ncbi.nlm.nih.gov/sra/

2
http://www.altanalyze.org

## Supplementary Material

Supplementary Figure 1 Decrease of early neurons in spina bifida *in utero*. Full scan spinal cords Immunohistochemistry for **(A)** Doublecortin (DCX) and **(B)** Tubulin β III showed a decrease in early neuron expression in MMC fetuses. In MMC spinal cords DCX and Tubulin β III is exposed and in contact with the amniotic fluid during gestation.
