# Supplementary figures and images for: Premature Neural Progenitor Cell Differentiation Into Astrocytes in Retinoic Acid-Induced Spina Bifida Rat Model
Source: Front Mol Neurosci. 2022 Jun 17;15:888351. doi: 10.3389/fnmol.2022.888351 (PMC9249056; doi:10.3389/fnmol.2022.888351)

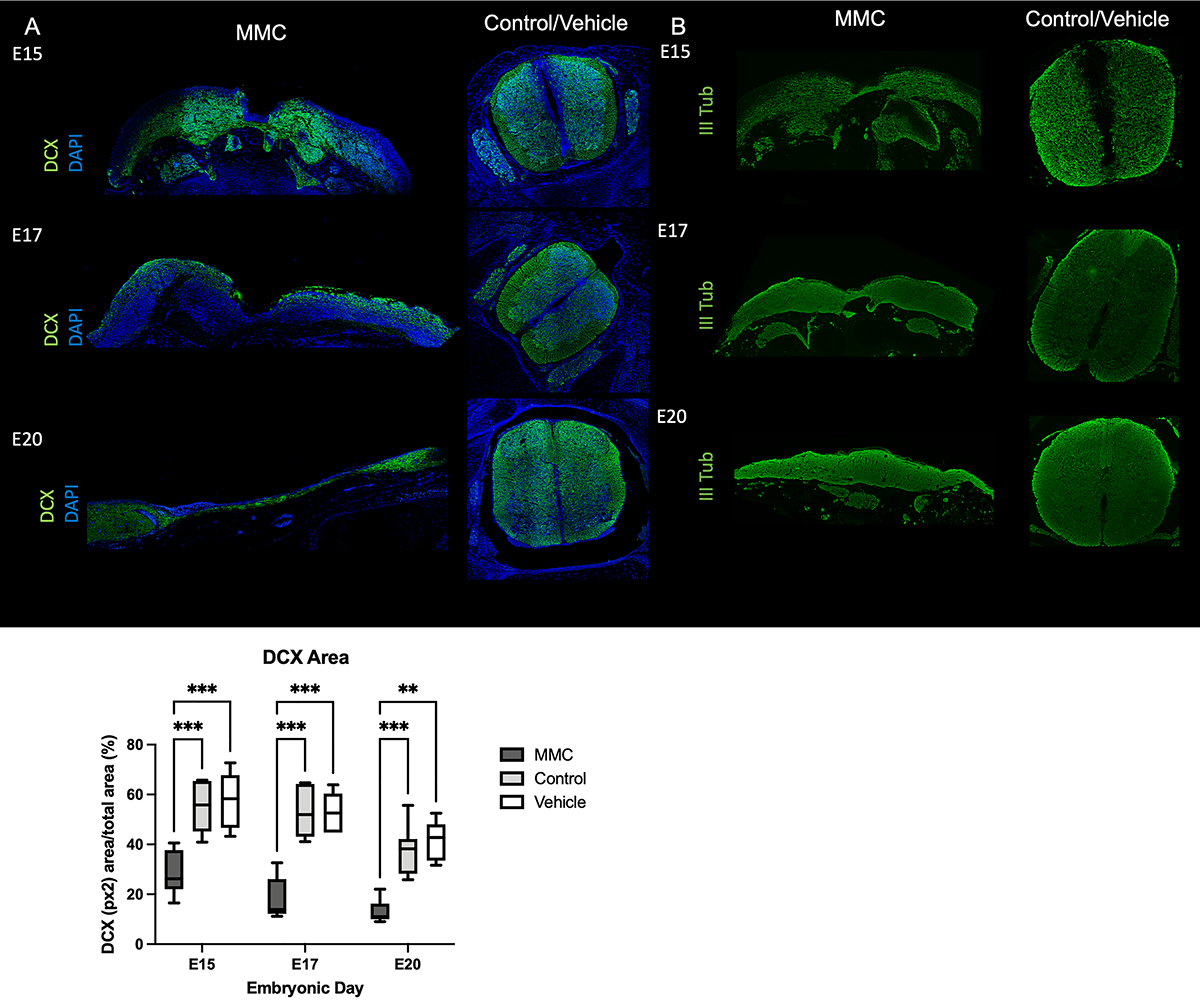

Supplement: Supplementary Figure 1 — Decrease of early neurons in spina bifida in utero. Full scan spinal cords Immunohistochemistry for (A) Doublecortin (DCX) and (B) Tubulin β III showed a decrease in early neuron expression in MMC fetuses. In MMC spinal cords DCX and Tubulin β III is exposed and in contact with the amniotic fluid during gestation. [file Image_1.TIFF]
