# Supplementary material for: Premature Neural Progenitor Cell Differentiation Into Astrocytes in Retinoic Acid-Induced Spina Bifida Rat Model
Source: Front Mol Neurosci. 2022 Jun 17;15:888351. doi: 10.3389/fnmol.2022.888351 (PMC9249056; doi:10.3389/fnmol.2022.888351)
Supplement: Supplementary file 2 [file Table_1.docx]

| **mRNA** | **Name** | ***Assay code number** |
| --- | --- | --- |
| Olig2 | Oligodendrocyte Lineage Transcription Factor 2 | Rn01767116_m1 |
| BMP2 | Bone Morphogenic Protein 2 | Rn00567818_m1 |
| BMP4 | Bone Morphogenic Protein 4 | Rn00432087_m1 |
| Pax6 | Paired Box 6 | Rn00689608_m1 |
| Sox9 | SRY-Box 9 | Rn01751070_m1 |
| Hprt1 | Hypoxanthine Phosphoribosyl Transferase 1 | Rn01527840_m1 |
| Nkx2.2 | Nk2 Homeobox 2 | Rn04244749_m1 |
| GFAP | Glial fibrillary acid protein | Rn01253033_m1 |

**Supplementary Table1**. TaqMan probes for gene expression assay

*Probes code assay from Thermo Fisher Scientific
