# Supplementary material for: Premature Neural Progenitor Cell Differentiation Into Astrocytes in Retinoic Acid-Induced Spina Bifida Rat Model
Source: Front Mol Neurosci. 2022 Jun 17;15:888351. doi: 10.3389/fnmol.2022.888351 (PMC9249056; doi:10.3389/fnmol.2022.888351)
Supplement: Supplementary file 3 [file Table_2.docx]

| E15 | | | | |
| --- | --- | --- | --- | --- |
|  | RNAseq | | RT-qPCR | |
| MMC | Gene | F-Change | Gene | F-Change |
|  | BMP2 | 1.30 | BMP2 | 3.85 |
|  | BMP4 | 1.98 | BMP4 | 4.15 |
|  | GFAP | 2.71 | GFAP | 2.07 |
|  | Nkx2.2 | 1.05 | Nkx2.2 | 1.80 |
|  | Pax6 | 1.36 | Pax6 | 1.84 |
|  | Sox9 | 1.9 | Sox9 | 2.38 |
|  | Olig2 | 0.28 | Olig2 | 0.41 |
| Control | UP | F-Change | UP | F-Change |
|  | BMP2 | 0.88 | BMP2 | 1.09 |
|  | BMP4 | 1.13 | BMP4 | 1.44 |
|  | GFAP | 1.61 | GFAP | 0.82 |
|  | Nkx2.2 | 1.22 | Nkx2.2 | 1.78 |
|  | Pax6 | 0.84 | Pax6 | 0.79 |
|  | Sox9 | 0.68 | Sox9 | 0.74 |
|  | Olig2 | 1.26 | Olig2 | 0.94 |
| E17 | | | | |
|  | RNAseq | | RT-qPCR | |
| MMC | Gene | F-Change | Gene | F-Change |
|  | BMP2 | 0.69 | BMP2 | 2.85 |
|  | BMP4 | 1.14 | BMP4 | 3.15 |
|  | GFAP | 1.25 | GFAP | 2.38 |
|  | Nkx2.2 | 1.61 | Nkx2.2 | 1.52 |
|  | Pax6 | 1.24 | Pax6 | 1.28 |
|  | Sox9 | 2.09 | Sox9 | 2.1 |
|  | Olig2 | 0.62 | Olig2 | 0.72 |
| Control | Gene | F-Change | Gene | F-Change |
|  | BMP2 | 1.08 | BMP2 | 0.92 |
|  | BMP4 | 0.86 | BMP4 | 1.27 |
|  | GFAP | 1.27 | GFAP | 1.00 |
|  | Nkx2.2 | 1.24 | Nkx2.2 | 1.14 |
|  | Pax6 | 1.06 | Pax6 | 0.87 |
|  | Sox9 | 1.19 | Sox9 | 1.00 |
|  | Olig2 | 1.14 | Olig2 | 1.06 |
| E20 | | | | |
|  | RNAseq | | RT-qPCR | |
| MMC | Gene | F-Change | Gene | F-Change |
|  | BMP2 | 0.98 | BMP2 | 0.82 |
|  | BMP4 | 2.43 | BMP4 | 0.92 |
|  | GFAP | 1.40 | GFAP | 2.83 |
|  | Nkx2.2 | 0.68 | Nkx2.2 | 1.38 |
|  | Pax6 | 0.35 | Pax6 | 1.01 |
|  | Sox9 | 1.55 | Sox9 | 0.97 |
| Control | Gene | F-Change | Gene | F-Change |
|  | BMP2 | 0.87 | BMP2 | 0.84 |
|  | BMP4 | 0.54 | BMP4 | 1.15 |
|  | GFAP | 1.45 | GFAP | 0.97 |
|  | Nkx2.2 | 1.07 | Nkx2.2 | 0.72 |
|  | Pax6 | 0.66 | Pax6 | 1.13 |
|  | Sox9 | 1.18 | Sox9 | 1.37 |
|  | Olig2 | 0.92 | Olig2 | 1.05 |
