# Supplementary material for: Premature Neural Progenitor Cell Differentiation Into Astrocytes in Retinoic Acid-Induced Spina Bifida Rat Model
Source: Front Mol Neurosci. 2022 Jun 17;15:888351. doi: 10.3389/fnmol.2022.888351 (PMC9249056; doi:10.3389/fnmol.2022.888351)
Supplement: Supplementary file 4 [file Table_3.docx]

| **MMC vs Vehicle E15**  **Total Genes: 346**  **Blue: Downregulated**  **Yellow: Upregulated** | | | | |
| --- | --- | --- | --- | --- |
| **Gene Name** | **Annotated term** | **Context** | **p-val** | **Fold Change** |
| **Abcc8** | Neurogenesis | negative regulation of glial cell proliferation | 0.001311 | 0.485620086 |
| **Ablim1** | Neurogenesis | axon guidance | 0.0010762 | 0.613701919 |
| **Acsl6** | Neurogenesis | neuroblast proliferation | 2.33E-07 | 0.423930597 |
|  |  | positive regulation of neuron projection development |  |  |
| **Adcy1** | Neurogenesis | axonogenesis | 1.83E-08 | 0.438880477 |
| **Adcyap1** | Neurogenesis | negative regulation of glial cell proliferation | 1.21E-06 | 0.451187811 |
|  |  | positive regulation of neuron projection development |  |  |
| **Agtpbp1** | Neurogenesis | cerebellar Purkinje cell differentiation | 7.81E-07 | 0.521948728 |
| **Ahi1** | Neurogenesis | positive regulation of neuron projection development | 0.00030725 | 0.619286971 |
| **Alcam** | Neurogenesis | motor neuron axon guidance | 0.0011695 | 0.643093316 |
| **Alk** | Neurogenesis | regulation of neuron differentiation | 0.0045692 | 0.623192597 |
| **Amigo1** | Neurogenesis | neuron projection fasciculation | 0.00016963 | 0.595994119 |
|  |  | positive regulation of axonogenesis |  |  |
|  |  | positive regulation of neuron projection development |  |  |
| **Apoe** | Neurogenesis | axon regeneration | 0.0015379 | 0.603326142 |
|  |  | positive regulation of dendritic spine development |  |  |
|  |  | positive regulation of neuron projection development |  |  |
| **Arhgap44** | Neurogenesis | regulation of dendritic spine morphogenesis | 0.0045199 | 0.632369635 |
| **Arhgef10** | Neurogenesis | myelination in peripheral nervous system | 0.00016386 | 1.731917249 |
| **Ascl1** | Neurogenesis | central nervous system neuron development | 5.72E-06 | 2.143249787 |
|  |  | commitment of neuronal cell to specific neuron type in forebrain |  |  |
|  |  | generation of neurons |  |  |
|  |  | glial cell differentiation |  |  |
|  |  | neuroblast fate determination |  |  |
|  |  | neuroblast proliferation |  |  |
|  |  | neuron fate commitment |  |  |
|  |  | neuron migration |  |  |
|  |  | oligodendrocyte cell fate commitment |  |  |
| **Aspm** | Neurogenesis | forebrain neuroblast division | 0.00028301 | 3.114930555 |
|  |  | negative regulation of neuron differentiation |  |  |
|  |  | neuron migration |  |  |
|  |  | positive regulation of neuroblast proliferation |  |  |
| **Astn1** | Neurogenesis | neuron migration | 0.00026954 | 0.630190539 |
| **Atcay** | Neurogenesis | neuron projection development | 3.72E-05 | 0.591799343 |
| **Atf1** | Neurogenesis | positive regulation of neuron projection development | 0.0010556 | 1.660065309 |
| **Atl1** | Neurogenesis | axonogenesis | 6.11E-05 | 0.564177095 |
| **Atp2b2** | Neurogenesis | auditory receptor cell stereocilium organization | 5.31E-08 | 0.466678206 |
|  |  | cerebellar granule cell differentiation |  |  |
|  |  | cerebellar Purkinje cell differentiation |  |  |
| **Aurka** | Neurogenesis | neuron projection extension | 5.39E-06 | 2.521531066 |
| **Axl** | Neurogenesis | neuron migration | 0.002053 | 1.545421099 |
| **Bcan** | Neurogenesis | neuron projection extension | 8.54E-14 | 0.098005057 |
| **Bcl11b** | Neurogenesis | axonogenesis | 0.0015234 | 0.545624375 |
|  |  | central nervous system neuron differentiation |  |  |
|  |  | commitment of neuronal cell to specific neuron type in forebrain |  |  |
| **Bcl11b** | Neurogenesis | regulation of neuron differentiation | 0.0015234 | 0.545624375 |
|  |  | striatal medium spiny neuron differentiation |  |  |
| **Bend6** | Neurogenesis | positive regulation of neuron differentiation | 5.84E-06 | 0.357397141 |
| **Boc** | Neurogenesis | axon guidance | 4.91E-08 | 2.096815611 |
| **Brinp1** | Neurogenesis | negative regulation of neurogenesis | 4.05E-08 | 0.445222991 |
|  |  | positive regulation of neuron differentiation |  |  |
| **Brinp2** | Neurogenesis | positive regulation of neuron differentiation | 6.14E-06 | 0.506737324 |
| **Brinp3** | Neurogenesis | positive regulation of neuron differentiation | 3.61E-05 | 0.391422882 |
| **Brsk1** | Neurogenesis | neuron differentiation | 0.00039025 | 0.642531906 |
|  |  | neuron projection morphogenesis |  |  |
|  |  | regulation of neuron projection development |  |  |
|  |  | regulation of axonogenesis |  |  |
| **Btg2** | Neurogenesis | central nervous system neuron development | 6.60E-07 | 1.981866796 |
|  |  | neuron differentiation |  |  |
|  |  | neuron projection development |  |  |
| **C1ql1** | Neurogenesis | neuron remodeling | 0.0014383 | 0.541453744 |
| **Cacng7** | Neurogenesis | positive regulation of dendrite extension | 0.00061497 | 0.624286425 |
| **Camk1d** | Neurogenesis | positive regulation of neuron projection development | 0.0003085 | 0.46998936 |
|  |  | regulation of dendrite development |  |  |
| **Camk2b** | Neurogenesis | positive regulation of dendritic spine morphogenesis | 4.41E-13 | 0.372290317 |
|  |  | regulation of neuron migration |  |  |
|  |  | positive regulation of neuron projection development |  |  |
| **Camsap3** | Neurogenesis | neuron projection development | 0.0011922 | 0.660939443 |
| **Casp6** | Neurogenesis | axonal fasciculation | 0.00046771 | 1.974175161 |
| **Cbln1** | Neurogenesis | cerebellar granule cell differentiation | 6.09E-06 | 0.554062258 |
| **Cck** | Neurogenesis | axonogenesis | 0.00024693 | 0.479731807 |
|  |  | neuron migration |  |  |
| **Cdc20** | Neurogenesis | regulation of dendrite development | 1.44E-07 | 3.318658087 |
| **Cdh1** | Neurogenesis | regulation of neuron migration | 7.50E-10 | 4.39694047 |
| **Cdh4** | Neurogenesis | positive regulation of axon extension | 4.46E-06 | 0.535214831 |
| **Cdk6** | Neurogenesis | gliogenesis | 0.0042467 | 2.044857061 |
| **Cdkn1c** | Neurogenesis | neuron maturation | 6.47E-07 | 2.00346874 |
| **Cdon** | Neurogenesis | positive regulation of neuron differentiation | 5.34E-12 | 2.886657614 |
| **Cend1** | Neurogenesis | cerebellar Purkinje cell differentiation | 4.30E-07 | 0.477310507 |
|  |  | neuron differentiation |  |  |
| **Chac1** | Neurogenesis | neurogenesis | 0.00015211 | 0.488354264 |
| **Chd5** | Neurogenesis | cerebral cortex neuron differentiation | 4.01E-13 | 0.295145858 |
| **Chodl** | Neurogenesis | positive regulation of axonogenesis | 1.46E-05 | 0.465257077 |
|  |  | regulation of neuron projection development |  |  |
| **Cit** | Neurogenesis | dendrite development | 0.0043 | 1.510231799 |
|  |  | negative regulation of neuron differentiation |  |  |
|  |  | negative regulation of dendrite morphogenesis |  |  |
| **Cnr1** | Neurogenesis | axonal fasciculation | 0.0001753 | 0.612401618 |
|  |  | positive regulation of neuron projection development |  |  |
| **Cntn1** | Neurogenesis | neuron projection development | 5.59E-08 | 0.457010748 |
|  |  | positive regulation of neuron projection development |  |  |
| **Cntn4** | Neurogenesis | axon guidance | 6.99E-12 | 0.307679451 |
|  |  | negative regulation of neuron differentiation |  |  |
|  |  | neuron projection development |  |  |
| **Cntn6** | Neurogenesis | axon guidance | 0.0026043 | 0.352623371 |
|  |  | dendrite self-avoidance |  |  |
|  |  | neuron differentiation |  |  |
| **Cntnap1** | Neurogenesis | central nervous system myelination | 0.0042014 | 0.590692828 |
|  |  | neuron projection development |  |  |
|  |  | neuron projection morphogenesis |  |  |
| **Col3a1** | Neurogenesis | negative regulation of neuron migration | 2.05E-06 | 2.03665272 |
| **Cpeb3** | Neurogenesis | positive regulation of dendritic spine development | 0.00046346 | 0.567074426 |
| **Cspg4** | Neurogenesis | glial cell migration | 0.0001251 | 1.755254224 |
|  |  | negative regulation of neuron projection development |  |  |
|  |  | neuron remodeling |  |  |
| **Cspg5** | Neurogenesis | glial cell projection elongation | 2.55E-05 | 0.58151165 |
| **Ctdsp1** | Neurogenesis | negative regulation of neuron differentiation | 0.00079874 | 1.58280113 |
|  |  | negative regulation of neurogenesis |  |  |
| **Ctnna1** | Neurogenesis | negative regulation of neuroblast proliferation | 0.00011362 | 1.640561867 |
| **Ctnna2** | Neurogenesis | axonogenesis | 0.00016172 | 0.613246925 |
|  |  | dendrite morphogenesis |  |  |
|  |  | regulation of neuron migration |  |  |
|  |  | regulation of neuron projection development |  |  |
| **Ctnnd2** | Neurogenesis | dendritic spine morphogenesis | 2.87E-06 | 0.55123523 |
| **Ctsz** | Neurogenesis | negative regulation of neuron projection development | 0.00090625 | 1.601040719 |
| **Cx3cl1** | Neurogenesis | microglial cell proliferation | 0.0022753 | 0.628846584 |
|  |  | positive regulation of neuroblast proliferation |  |  |
|  |  | positive regulation of neuron projection development |  |  |
| **Dclk1** | Neurogenesis | axonogenesis | 0.00026077 | 0.629684037 |
|  |  | central nervous system projection neuron axonogenesis |  |  |
|  |  | neuron migration |  |  |
| **Dcx** | Neurogenesis | axon extension | 5.72E-06 | 0.569856273 |
|  |  | central nervous system projection neuron axonogenesis |  |  |
|  |  | dendrite morphogenesis |  |  |
|  |  | layer formation in cerebral cortex |  |  |
|  |  | neuron migration |  |  |
| **Dlg2** | Neurogenesis | neuronal ion channel clustering | 4.45E-06 | 0.423578128 |
| **Dlg4** | Neurogenesis | dendritic spine morphogenesis | 9.68E-07 | 0.539838584 |
|  |  | positive regulation of neuron projection arborization |  |  |
| **Dll1** | Neurogenesis | negative regulation of neuron differentiation | 3.08E-08 | 2.085509668 |
| **Dll3** | Neurogenesis | negative regulation of neurogenesis | 0.0014602 | 1.825497888 |
| **Dmrta2** | Neurogenesis | dopaminergic neuron differentiation | 0.0045734 | 2.281053051 |
|  |  | positive regulation of neuroblast proliferation |  |  |
| **Dner** | Neurogenesis | glial cell differentiation | 3.05E-06 | 0.552210414 |
| **Dnm3** | Neurogenesis | negative regulation of dendritic spine morphogenesis | 0.00016109 | 0.585304794 |
| **Dok5** | Neurogenesis | neuron differentiation | 0.0027378 | 0.621880808 |
| **Dpysl3** | Neurogenesis | negative regulation of neuron projection development | 1.95E-05 | 0.593672833 |
| **Draxin** | Neurogenesis | axon guidance | 0.00070404 | 0.604485651 |
|  |  | commissural neuron differentiation in spinal cord |  |  |
|  |  | negative regulation of axon extension |  |  |
| **Drd2** | Neurogenesis | axonogenesis | 0.0047962 | 0.510311479 |
|  |  | cerebral cortex GABAergic interneuron migration |  |  |
|  |  | positive regulation of neuroblast proliferation |  |  |
| **Dscam** | Neurogenesis | dendrite morphogenesis | 0.00015651 | 0.39033913 |
|  |  | positive regulation of axon extension involved in axon guidance |  |  |
| **Dscaml1** | Neurogenesis | axon guidance | 1.17E-12 | 0.365615718 |
|  |  | dendrite self-avoidance |  |  |
| **E2f1** | Neurogenesis | positive regulation of glial cell proliferation | 1.42E-08 | 2.325111658 |
| **Ect2** | Neurogenesis | positive regulation of neuron differentiation | 1.21E-07 | 2.459389097 |
| **Eef1a1** | Neurogenesis | positive regulation of neuron projection development | 0.00055811 | 1.533119337 |
| **Efemp1** | Neurogenesis | negative regulation of neuron projection development | 0.002924 | 1.870015204 |
|  |  | regulation of glial cell migration |  |  |
| **Efna4** | Neurogenesis | axon guidance | 1.82E-05 | 2.008891992 |
| **Efnb3** | Neurogenesis | axon choice point recognition | 4.44E-06 | 0.565277038 |
|  |  | negative regulation of axonogenesis |  |  |
| **Egflam** | Neurogenesis | dendrite development | 0.00046324 | 1.623908111 |
|  |  | motor neuron axon guidance |  |  |
| **Egfr** | Neurogenesis | neuron projection morphogenesis | 9.79E-05 | 3.767480383 |
| **Egr2** | Neurogenesis | motor neuron axon guidance | 1.05E-06 | 2.681425183 |
|  |  | Schwann cell differentiation |  |  |
| **Enc1** | Neurogenesis | positive regulation of neuron projection development | 1.83E-05 | 0.59588672 |
| **Epha2** | Neurogenesis | neuron differentiation | 0.0019142 | 2.066945 |
| **Epha3** | Neurogenesis | axon guidance | 1.12E-05 | 2.118289836 |
|  |  | regulation of neuron projection development |  |  |
| **Epha5** | Neurogenesis | axon guidance | 1.86E-06 | 0.51462377 |
|  |  | dendritic spine morphogenesis |  |  |
| **Epha8** | Neurogenesis | axon guidance | 1.26E-05 | 0.459138081 |
|  |  | neuron projection development |  |  |
|  |  | neuron remodeling |  |  |
| **Ephb1** | Neurogenesis | axon guidance | 0.00017827 | 0.625382173 |
| **Erbb2** | Neurogenesis | glial cell differentiation | 3.07E-09 | 2.481650587 |
|  |  | oligodendrocyte differentiation |  |  |
|  |  | motor neuron axon guidance |  |  |
| **Esrp1** | Neurogenesis | regulation of inner ear auditory receptor cell fate specification | 3.69E-09 | 23.69482335 |
| **Etv6** | Neurogenesis | neurogenesis | 0.0046953 | 1.743903246 |
| **Ezh2** | Neurogenesis | positive regulation of dendrite development | 0.0015363 | 1.517819253 |
|  |  | regulation of gliogenesis |  |  |
|  |  | regulation of neurogenesis |  |  |
| **Faim2** | Neurogenesis | cerebellar Purkinje cell differentiation | 2.01E-08 | 0.292052573 |
| **Fez1** | Neurogenesis | positive regulation of neuron differentiation | 0.00050141 | 0.642638803 |
|  |  | positive regulation of neuron projection development |  |  |
| **Fgf13** | Neurogenesis | establishment of neuroblast polarity | 2.17E-05 | 0.494142826 |
|  |  | negative regulation of collateral sprouting |  |  |
|  |  | neuron migration |  |  |
| **Fgfr2** | Neurogenesis | axonogenesis | 0.0015105 | 1.822918422 |
|  |  | pyramidal neuron development |  |  |
|  |  | ventricular zone neuroblast division |  |  |
| **Flna** | Neurogenesis | negative regulation of neuron projection development | 0.00046503 | 2.686448162 |
|  |  | positive regulation of neuron migration |  |  |
| **Fn1** | Neurogenesis | glial cell migration | 1.79E-05 | 2.921076214 |
|  |  | positive regulation of axon extension |  |  |
| **Foxd1** | Neurogenesis | axon guidance | 2.21E-07 | 3.040903427 |
| **Gabra5** | Neurogenesis | inner ear receptor cell development | 1.70E-10 | 0.289131961 |
|  |  | neuron development |  |  |
| **Gabrb3** | Neurogenesis | inner ear receptor cell development | 1.14E-10 | 0.404496745 |
|  |  | neuron development |  |  |
| **Gap43** | Neurogenesis | axon choice point recognition | 2.81E-05 | 0.587666677 |
|  |  | glial cell differentiation |  |  |
|  |  | axon guidance |  |  |
| **Gas1** | Neurogenesis | axon guidance | 1.21E-15 | 3.675651534 |
| **Gfap** | Neurogenesis | negative regulation of neuron projection development | 0.0040694 | 2.714525431 |
|  |  | neuron projection regeneration |  |  |
|  |  | positive regulation of glial cell proliferation |  |  |
|  |  | positive regulation of Schwann cell proliferation |  |  |
| **Gfra3** | Neurogenesis | axon guidance | 0.0011155 | 1.687350869 |
|  |  | neuron development |  |  |
|  |  | neuron migration |  |  |
| **Gli2** | Neurogenesis | axon guidance | 0.00022481 | 2.739855866 |
|  |  | neuron development |  |  |
|  |  | positive regulation of neuron differentiation |  |  |
|  |  | smoothened signaling pathway involved in spinal cord motor neuron cell fate specification |  |  |
|  |  | spinal cord motor neuron differentiation |  |  |
|  |  | spinal cord ventral commissure morphogenesis |  |  |
| **Gpm6a** | Neurogenesis | neuron migration | 5.64E-10 | 0.45087518 |
|  |  | neuron projection development |  |  |
|  |  | neuron projection morphogenesis |  |  |
| **Grid2** | Neurogenesis | cerebellar granule cell differentiation | 4.42E-05 | 0.459806893 |
|  |  | regulation of neuron projection development |  |  |
| **Grin1** | Neurogenesis | positive regulation of Schwann cell migration | 1.45E-14 | 0.307210621 |
|  |  | regulation of axonogenesis |  |  |
| **Grin3a** | Neurogenesis | dendrite development | 9.74E-07 | 0.199256681 |
| **Grip2** | Neurogenesis | positive regulation of neuron maturation | 7.64E-08 | 0.402343603 |
| **Grm5** | Neurogenesis | positive regulation of long-term neuronal synaptic plasticity | 3.31E-30 | 0.114832268 |
| **Gsx1** | Neurogenesis | neuron fate commitment | 0.0039313 | 2.498738626 |
|  |  | spinal cord association neuron differentiation |  |  |
| **Gsx2** | Neurogenesis | neuron fate commitment | 3.64E-05 | 2.398279828 |
|  |  | neuron fate specification |  |  |
|  |  | positive regulation of oligodendrocyte differentiation |  |  |
|  |  | spinal cord association neuron differentiation |  |  |
|  |  | subpallium neuron fate commitment |  |  |
| **Hdac1** | Neurogenesis | neuron differentiation | 8.32E-05 | 1.66987487 |
|  |  | positive regulation of oligodendrocyte differentiation |  |  |
| **Hdac11** | Neurogenesis | oligodendrocyte development | 0.002691 | 0.549842375 |
| **Hes1** | Neurogenesis | cell morphogenesis involved in neuron differentiation | 1.14E-08 | 2.331405575 |
|  |  | forebrain radial glial cell differentiation |  |  |
|  |  | negative regulation of neuron differentiation |  |  |
|  |  | negative regulation of oligodendrocyte differentiation |  |  |
|  |  | regulation of neurogenesis |  |  |
| **Hes5** | Neurogenesis | central nervous system myelination | 2.27E-07 | 3.063115994 |
|  |  | forebrain radial glial cell differentiation |  |  |
|  |  | glial cell fate commitment |  |  |
|  |  | negative regulation of neuron differentiation |  |  |
|  |  | negative regulation of neuron differentiation |  |  |
|  |  | negative regulation of oligodendrocyte differentiation |  |  |
| **Hes6** | Neurogenesis | regulation of neurogenesis | 3.28E-05 | 1.694758845 |
| **Heyl** | Neurogenesis | positive regulation of neuron differentiation | 2.19E-07 | 2.41295331 |
| **Hmg20b** | Neurogenesis | positive regulation of neuron differentiation | 1.49E-05 | 1.886824187 |
| **Hmgb2** | Neurogenesis | regulation of neurogenesis | 6.76E-15 | 2.699327454 |
| **Hoxc10** | Neurogenesis | spinal cord motor neuron cell fate specification | 0.0028315 | 2.42738004 |
| **Hoxc8** | Neurogenesis | neuron differentiation | 1.00E-14 | 0.169317148 |
| **Hspa5** | Neurogenesis | positive regulation of neuron projection development | 0.00060421 | 1.536087076 |
| **Hspb1** | Neurogenesis | positive regulation of neuron projection development | 2.67E-07 | 3.130948981 |
| **Htr7** | Neurogenesis | positive regulation of neuron projection development | 0.0032171 | 0.509636319 |
| **Id1** | Neurogenesis | negative regulation of dendrite morphogenesis | 0.0051217 | 1.596264804 |
| **Id3** | Neurogenesis | negative regulation of neuron differentiation | 5.35E-05 | 1.708168151 |
| **Idh2** | Neurogenesis | negative regulation of glial cell migration | 2.90E-05 | 1.908673057 |
| **Ier2** | Neurogenesis | neuron differentiation | 0.0015572 | 1.944406686 |
| **Igf1** | Neurogenesis | glial cell differentiation and proliferation | 2.95E-09 | 3.085490815 |
| **Igsf10** | Neurogenesis | regulation of neuron migration | 3.23E-10 | 2.548767896 |
| **Inhba** | Neurogenesis | GABAergic neuron differentiation | 0.001966 | 3.507395207 |
|  |  | striatal medium spiny neuron differentiation |  |  |
| **Inpp5j** | Neurogenesis | negative regulation of neuron projection development | 0.0011855 | 0.473192791 |
| **Inppl1** | Neurogenesis | negative regulation of neuron projection development | 2.21E-05 | 1.80962404 |
| **Irx5** | Neurogenesis | neuron maturation | 0.0029997 | 1.992307037 |
| **Itga3** | Neurogenesis | neuron migration | 0.00020368 | 0.581358502 |
|  |  | positive regulation of neuron projection development |  |  |
| **Itga6** | Neurogenesis | positive regulation of neuron projection development | 0.0025224 | 1.518219093 |
| **Kcnma1** | Neurogenesis | inner ear auditory receptor cell differentiation | 8.46E-06 | 0.489540458 |
| **Kidins220** | Neurogenesis | dendrite morphogenesis | 0.00036355 | 0.61828761 |
|  |  | positive regulation of neuron projection development |  |  |
| **Kif20b** | Neurogenesis | neuron projection morphogenesis | 0.00023169 | 2.295486584 |
|  |  | positive regulation of neuron migration |  |  |
| **Kif3a** | Neurogenesis | axon guidance | 9.91E-05 | 0.60178915 |
| **Kif5c** | Neurogenesis | axon guidance | 0.0024859 | 0.661191462 |
| **Kirrel3** | Neurogenesis | neuron migration | 3.21E-05 | 0.549057824 |
|  |  | neuron projection morphogenesis |  |  |
| **Kit** | Neurogenesis | positive regulation of long-term neuronal synaptic plasticity | 3.03E-05 | 0.566328092 |
| **Klf15** | Neurogenesis | glial cell differentiation | 0.00069433 | 0.441045683 |
| **Klf4** | Neurogenesis | regulation of axon regeneration | 0.0015457 | 2.214222191 |
| **Klhl1** | Neurogenesis | dendrite development | 0.0036813 | 0.540231624 |
| **Kndc1** | Neurogenesis | cerebellar granule cell differentiation | 2.72E-16 | 0.267201498 |
|  |  | regulation of dendrite development |  |  |
| **L1cam** | Neurogenesis | axon guidance | 0.0022196 | 0.618381901 |
|  |  | axonal fasciculation |  |  |
|  |  | neuron projection development |  |  |
| **Lamb1** | Neurogenesis | neuron projection development | 0.00030053 | 1.574332204 |
|  |  | neuronal-glial interaction involved in cerebral cortex radial glia guided migration |  |  |
| **Lamc1** | Neurogenesis | neuron projection development | 2.02E-05 | 1.714989805 |
| **Lamc2** | Neurogenesis | dendrite development | 7.66E-05 | 3.59352569 |
|  |  | motor neuron axon guidance |  |  |
| **Lef1** | Neurogenesis | forebrain neuroblast division | 2.27E-05 | 2.01042428 |
|  |  | forebrain radial glial cell differentiation |  |  |
| **Lgals1** | Neurogenesis | negative regulation of neuron projection development | 2.05E-07 | 2.078582485 |
| **Lgi1** | Neurogenesis | axon guidance | 1.38E-05 | 0.275228161 |
|  | Neurogenesis | neuron projection development | 1.38E-05 | 0.275228161 |
| **Lgi4** | Neurogenesis | glial cell development | 1.27E-06 | 3.292309579 |
| **Lmx1b** | Neurogenesis | central nervous system neuron development | 2.45E-09 | 0.433769344 |
|  |  | neuron migration |  |  |
| **Lpar1** | Neurogenesis | negative regulation of neuron projection development | 0.00041722 | 1.675509655 |
|  |  | positive regulation of dendritic spine development |  |  |
| **Lrp8** | Neurogenesis | dendrite morphogenesis | 0.00058592 | 0.648190598 |
|  |  | layer formation in cerebral cortex |  |  |
|  |  | positive regulation of dendrite development |  |  |
| **Lrrc7** | Neurogenesis | positive regulation of neuron projection development | 0.0020642 | 0.259984947 |
| **Lrtm2** | Neurogenesis | axon guidance | 4.27E-09 | 0.292883739 |
| **Lzts3** | Neurogenesis | regulation of dendritic spine morphogenesis | 0.00010704 | 0.559352635 |
| **Mag** | Neurogenesis | axon regeneration | 0.0021043 | 0.255943984 |
|  |  | central nervous system myelination |  |  |
|  |  | negative regulation of axon extension |  |  |
|  |  | negative regulation of neuron differentiation |  |  |
|  |  | negative regulation of neuron projection development |  |  |
| **Magi2** | Neurogenesis | positive regulation of neuron projection development | 1.05E-09 | 0.4280348 |
| **Mapk8** | Neurogenesis | dendrite morphogenesis | 0.00030905 | 0.618030525 |
|  |  | neuron migration |  |  |
|  |  | neuron projection development |  |  |
|  |  | positive regulation of neuroblast proliferation |  |  |
| **Mapk9** | Neurogenesis | neuron projection development | 0.0018928 | 0.622057566 |
| **Mapt** | Neurogenesis | axon extension | 2.86E-12 | 0.411253539 |
|  |  | axonogenesis |  |  |
|  |  | neuron migration |  |  |
|  |  | positive regulation of neuron projection development |  |  |
| **Mark1** | Neurogenesis | neuron migration | 0.001184 | 0.6491123 |
|  |  | regulation of dendrite development |  |  |
|  |  | regulation of neuron projection development |  |  |
| **Mdk** | Neurogenesis | glial cell projection elongation | 6.12E-14 | 2.599798842 |
|  |  | positive regulation of neuron migration |  |  |
|  |  | positive regulation of neuron projection development |  |  |
|  |  | positive regulation of oligodendrocyte differentiation |  |  |
| **Mef2c** | Neurogenesis | cell morphogenesis involved in neuron differentiation | 0.0020083 | 1.628698996 |
|  |  | neuron development |  |  |
|  |  | neuron migration |  |  |
|  |  | positive regulation of neuron differentiation |  |  |
|  |  | regulation of dendritic spine development |  |  |
| **Metrn** | Neurogenesis | glial cell differentiation | 9.64E-06 | 1.803275719 |
|  |  | positive regulation of axonogenesis |  |  |
| **Mgll** | Neurogenesis | regulation of axon extension | 0.0046544 | 0.595700882 |
| **Mmd2** | Neurogenesis | positive regulation of neuron differentiation | 0.00059269 | 0.453885404 |
| **Mmp2** | Neurogenesis | peripheral nervous system axon regeneration | 7.30E-05 | 2.016145838 |
| **Mmp24** | Neurogenesis | glial cell differentiation | 1.18E-05 | 0.574600041 |
| **Mov10** | Neurogenesis | regulation of neuron projection arborization | 3.88E-05 | 2.058223998 |
| **Mt3** | Neurogenesis | negative regulation of neurogenesis | 7.90E-08 | 0.369283319 |
| **Mturn** | Neurogenesis | neuron development | 8.47E-06 | 0.546021627 |
| **Myo16** | Neurogenesis | neuron projection morphogenesis | 2.94E-05 | 0.576578921 |
| **Myt1l** | Neurogenesis | neuron development | 0.00074315 | 0.377513463 |
|  |  | neuron differentiation |  |  |
|  |  | neuron fate commitment |  |  |
|  |  | neuron fate specification |  |  |
| **Nab2** | Neurogenesis | Schwann cell differentiation | 5.59E-11 | 2.854424691 |
| **Nap1l1** | Neurogenesis | positive regulation of neurogenesis | 0.00078155 | 1.52536024 |
| **Nap1l2** | Neurogenesis | positive regulation of neuron differentiation | 6.93E-12 | 0.36948815 |
| **Ncam1** | Neurogenesis | axonal fasciculation | 0.0003108 | 0.639789846 |
|  |  | commissural neuron axon guidance |  |  |
|  |  | neuron development |  |  |
|  |  | neuron projection development |  |  |
| **Nde1** | Neurogenesis | neuroblast proliferation | 4.83E-06 | 2.399111152 |
|  |  | neuron migration |  |  |
| **Ndrg4** | Neurogenesis | positive regulation of neuron projection development | 3.72E-07 | 0.515512745 |
| **Nefh** | Neurogenesis | peripheral nervous system neuron axonogenesis | 7.15E-06 | 0.468948045 |
| **Nefl** | Neurogenesis | neuron projection morphogenesis | 0.00087404 | 0.660714999 |
|  |  | peripheral nervous system axon regeneration |  |  |
|  |  | positive regulation of axonogenesis |  |  |
|  |  | regulation of axon diameter |  |  |
| **Negr1** | Neurogenesis | positive regulation of neuron projection development | 5.95E-09 | 0.351744556 |
| **Neurod4** | Neurogenesis | amacrine cell differentiation | 0.00083011 | 3.592529492 |
|  |  | neuron migration |  |  |
| **Neurog1** | Neurogenesis | neurogenesis | 0.0012056 | 7.512536004 |
|  |  | positive regulation of neuron differentiation |  |  |
|  |  | regulation of neuron differentiation |  |  |
| **Neurog3** | Neurogenesis | positive regulation of neuron differentiation | 0.0052186 | 4.248047801 |
|  |  | regulation of dendrite morphogenesis |  |  |
| **Nexn** | Neurogenesis | axon guidance | 3.99E-06 | 3.983951267 |
|  |  | dendrite self-avoidance |  |  |
| **Nfatc4** | Neurogenesis | negative regulation of dendrite morphogenesis | 8.17E-09 | 2.310812173 |
| **Nlgn1** | Neurogenesis | negative regulation of dendritic spine morphogenesis | 0.00041098 | 0.415984016 |
|  |  | neuron projection development |  |  |
|  |  | positive regulation of dendritic spine development |  |  |
|  |  | regulation of neuron differentiation |  |  |
| **Notch1** | Neurogenesis | auditory receptor cell fate commitment | 0.00059529 | 2.178145003 |
|  |  | axonogenesis |  |  |
|  |  | regulation of glial cell proliferation |  |  |
|  |  | negative regulation of neuron differentiation |  |  |
|  |  | negative regulation of oligodendrocyte differentiation |  |  |
| **Nptx1** | Neurogenesis | axonogenesis involved in innervation | 1.46E-05 | 0.440831738 |
| **Nptxr** | Neurogenesis | neuron projection development | 0.0019767 | 0.527184552 |
| **Nrg3** | Neurogenesis | negative regulation of neuron migration | 4.99E-05 | 0.290759856 |
| **Nrk** | Neurogenesis | neuron projection morphogenesis | 1.59E-16 | 9.40546975 |
| **Nrn1** | Neurogenesis | axonogenesis | 0.00015507 | 0.556093739 |
|  |  | neuron projection extension |  |  |
| **Nrxn1** | Neurogenesis | cerebellar granule cell differentiation | 0.0022051 | 0.416099367 |
|  |  | neuron maturation |  |  |
|  |  | neuron projection development |  |  |
| **Nsmf** | Neurogenesis | positive regulation of neuron migration | 6.46E-06 | 0.546392657 |
|  |  | regulation of dendrite morphogenesis |  |  |
| **Ntm** | Neurogenesis | negative regulation of neuron projection development | 2.76E-06 | 0.521471387 |
| **Ntrk2** | Neurogenesis | central nervous system neuron development | 8.38E-07 | 0.527978102 |
|  |  | mechanoreceptor differentiation |  |  |
|  |  | myelination in peripheral nervous system |  |  |
|  |  | neuron differentiation |  |  |
|  |  | neuron migration |  |  |
|  |  | oligodendrocyte differentiation |  |  |
|  |  | peripheral nervous system neuron development |  |  |
|  |  | positive regulation of axonogenesis |  |  |
|  |  | positive regulation of neuron projection development |  |  |
|  |  | regulation of dendrite development |  |  |
| **Ntrk3** | Neurogenesis | mechanoreceptor differentiation | 0.00020822 | 0.562038163 |
|  |  | myelination in peripheral nervous system |  |  |
|  |  | neuron fate specification |  |  |
|  |  | neuron migration |  |  |
|  |  | positive regulation of axon extension involved in regeneration |  |  |
|  |  | positive regulation of neuron projection development |  |  |
| **Nup133** | Neurogenesis | neurogenesis | 0.0012854 | 1.606420962 |
| **Olfm1** | Neurogenesis | negative regulation of neuron migration | 2.50E-06 | 0.550238884 |
|  |  | regulation of axon extension |  |  |
| **Olfm3** | Neurogenesis | eye photoreceptor cell development | 7.56E-11 | 0.308640652 |
| **Olig1** | Neurogenesis | neuron fate commitment | 0.00030213 | 0.25273546 |
|  |  | oligodendrocyte differentiation |  |  |
| **Olig2** | Neurogenesis | negative regulation of neuron differentiation | 2.14E-10 | 0.281576727 |
|  |  | oligodendrocyte differentiation |  |  |
|  |  | spinal cord oligodendrocyte cell fate specification |  |  |
| **Omg** | Neurogenesis | central nervous system myelination | 0.0019134 | 0.32371587 |
|  |  | neuron projection regeneration |  |  |
|  |  | regulation of collateral sprouting of intact axon in response to injury |  |  |
| **Oprm1** | Neurogenesis | positive regulation of neurogenesis | 7.39E-05 | 0.129040872 |
| **P2ry12** | Neurogenesis | cytosolic calcium signaling involved in initiation of cell movement in glial-mediated radial cell migration | 0.0030273 | 0.317427715 |
|  |  | positive regulation of microglial cell migration |  |  |
| **Pacsin1** | Neurogenesis | neuron development | 2.21E-05 | 0.434883236 |
|  |  | neuron projection morphogenesis |  |  |
|  |  | positive regulation of dendrite development |  |  |
| **Pak3** | Neurogenesis | positive regulation of dendritic spine morphogenesis | 0.0041673 | 0.515194822 |
|  |  | regulation of axonogenesis |  |  |
|  |  | regulation of neuron projection development |  |  |
| **Pak4** | Neurogenesis | dendritic spine development | 0.0011688 | 1.71422918 |
| **Palld** | Neurogenesis | neuron projection development | 0.00010432 | 2.039195366 |
| **Pax3** | Neurogenesis | neuron fate commitment | 0.0016631 | 2.144884556 |
|  |  | spinal cord association neuron differentiation |  |  |
| **Pcp4** | Neurogenesis | positive regulation of neuron differentiation | 2.52E-05 | 0.468395785 |
|  |  | positive regulation of neuron projection development |  |  |
| **Phactr1** | Neurogenesis | dendrite arborization | 0.0002411 | 0.590635509 |
|  |  | regulation of neuron migration |  |  |
| **Pitx2** | Neurogenesis | neuron migration | 5.43E-07 | 3.336649163 |
| **Pitx3** | Neurogenesis | dopaminergic neuron differentiation | 3.64E-18 | 41.73540888 |
|  |  | negative regulation of gliogenesis |  |  |
|  |  | negative regulation of neurogenesis |  |  |
|  |  | neuron development |  |  |
| **Plp1** | Neurogenesis | axon development | 3.15E-05 | 2.097687834 |
|  |  | central nervous system myelination |  |  |
|  |  | glial cell differentiation |  |  |
|  |  | neuron projection development |  |  |
| **Plxnc1** | Neurogenesis | positive regulation of axonogenesis | 0.00012041 | 0.584866799 |
|  |  | semaphorin-plexin signaling pathway involved in axon guidance |  |  |
| **Plxnd1** | Neurogenesis | positive regulation of axonogenesis | 1.39E-05 | 1.805814874 |
|  |  | semaphorin-plexin signaling pathway involved in axon guidance |  |  |
| **Postn** | Neurogenesis | neuron projection extension | 2.06E-19 | 6.316388897 |
| **Ppp2r3a** | Neurogenesis | eye photoreceptor cell differentiation | 0.001003 | 0.610388633 |
| **Ppp2r5b** | Neurogenesis | positive regulation of neuron projection development | 0.0053976 | 0.664582404 |
| **Ppp3r1** | Neurogenesis | myelination in peripheral nervous system | 0.00092693 | 0.652576979 |
|  |  | Schwann cell development |  |  |
| **Prdm12** | Neurogenesis | neurogenesis | 2.19E-08 | 2.199537198 |
|  |  | neuron projection development |  |  |
| **Prdm13** | Neurogenesis | neurogenesis | 7.81E-06 | 2.464337753 |
| **Prickle2** | Neurogenesis | neuron projection development | 0.0002993 | 0.614894156 |
| **Prkca** | Neurogenesis | central nervous system neuron axonogenesis | 0.0011438 | 0.598660505 |
| **Prkcz** | Neurogenesis | neuron projection extension | 1.25E-05 | 0.548681181 |
| **Prrx1** | Neurogenesis | neuron fate determination | 1.65E-12 | 3.296876854 |
|  |  | regulation of neuron projection regeneration |  |  |
| **Psd** | Neurogenesis | neuron projection development | 1.11E-13 | 0.370103325 |
| **Ptbp1** | Neurogenesis | negative regulation of neuron differentiation | 3.81E-06 | 2.27647245 |
|  |  | positive regulation of neuron projection development |  |  |
| **Ptf1a** | Neurogenesis | amacrine cell differentiation | 6.11E-08 | 3.255998421 |
|  |  | generation of neurons |  |  |
|  |  | neuron fate commitment |  |  |
| **Ptk7** | Neurogenesis | positive regulation of neuron projection development | 1.55E-05 | 1.891131913 |
| **Ptpn5** | Neurogenesis | positive regulation of neuron projection development | 5.28E-09 | 0.456251118 |
| **Ptpro** | Neurogenesis | axon guidance | 5.66E-15 | 0.358613076 |
| **Rab3a** | Neurogenesis | axonogenesis | 1.28E-06 | 0.533710759 |
| **Rac3** | Neurogenesis | cerebral cortex GABAergic interneuron development | 1.43E-07 | 0.484007061 |
|  |  | neuron projection development |  |  |
|  |  | regulation of neuron maturation |  |  |
| **Rap1gap** | Neurogenesis | negative regulation of neuron differentiation | 1.43E-05 | 0.544287195 |
| **Rasgrf1** | Neurogenesis | neuron projection development | 0.0042939 | 0.239351043 |
| **Rhobtb3** | Neurogenesis | motor neuron axon guidance | 8.02E-07 | 1.958922063 |
| **Rhog** | Neurogenesis | motor neuron axon guidance | 0.00025668 | 1.729362127 |
| **Rims1** | Neurogenesis | positive regulation of dendrite extension | 6.60E-07 | 0.475692109 |
| **Rit2** | Neurogenesis | regulation of neuron projection development | 8.56E-09 | 0.380534721 |
| **Rnf112** | Neurogenesis | neuron differentiation | 5.99E-05 | 0.504837155 |
| **Rnf157** | Neurogenesis | positive regulation of dendrite extension | 5.52E-05 | 0.586019266 |
| **Robo2** | Neurogenesis | axon midline choice point recognition | 0.00095079 | 0.580074458 |
|  |  | positive regulation of axonogenesis |  |  |
|  |  | retinal ganglion cell axon guidance |  |  |
| **Ror2** | Neurogenesis | positive regulation of neuron projection development | 5.78E-10 | 2.403272099 |
| **Rufy3** | Neurogenesis | regulation of axonogenesis | 2.53E-05 | 0.581926964 |
| **Runx2** | Neurogenesis | neuron differentiation | 3.23E-07 | 7.060645089 |
| **Runx3** | Neurogenesis | axon guidance | 4.93E-05 | 2.27599912 |
|  |  | neuron differentiation |  |  |
|  |  | neuron projection development |  |  |
| **Samd14** | Neurogenesis | neuron projection development | 6.48E-06 | 0.564940173 |
| **Sdc2** | Neurogenesis | dendrite morphogenesis | 0.001383 | 1.590929604 |
| **Sdc4** | Neurogenesis | inner ear receptor cell stereocilium organization | 0.0014425 | 2.149051417 |
| **Sema3c** | Neurogenesis | axon guidance | 0.00027384 | 0.620610495 |
|  |  | negative regulation of axon extension involved in axon guidance |  |  |
| **Sema4f** | Neurogenesis | axon guidance | 0.00078751 | 0.622204183 |
|  |  | negative regulation of axon extension involved in axon guidance |  |  |
| **Sema4g** | Neurogenesis | negative regulation of axon extension involved in axon guidance | 9.35E-07 | 0.509692843 |
| **Serpinf1** | Neurogenesis | positive regulation of neurogenesis | 0.0011198 | 1.72584153 |
|  |  | positive regulation of neuron projection development |  |  |
| **Serpini1** | Neurogenesis | positive regulation of neuron projection development | 0.00030612 | 0.532620551 |
| **Sez6** | Neurogenesis | regulation of dendrite development | 1.15E-05 | 0.556764835 |
| **Sfrp2** | Neurogenesis | regulation of midbrain dopaminergic neuron differentiation | 1.48E-07 | 4.085464171 |
|  |  | regulation of neuron projection development |  |  |
| **Sh3gl2** | Neurogenesis | dendrite extension | 8.51E-11 | 0.357719334 |
|  |  | neuron projection development |  |  |
| **Shank1** | Neurogenesis | dendritic spine morphogenesis | 6.44E-10 | 0.445840629 |
|  |  | positive regulation of dendritic spine development |  |  |
| **Shank2** | Neurogenesis | dendritic spine morphogenesis | 0.0015768 | 0.563766634 |
|  |  | positive regulation of dendritic spine development |  |  |
| **Shc1** | Neurogenesis | neuron differentiation | 1.47E-07 | 2.019222652 |
|  |  | neuron projection development |  |  |
| **Six1** | Neurogenesis | regulation of neuron differentiation | 4.61E-11 | 3.0763081 |
| **Six4** | Neurogenesis | generation of neurons | 1.04E-07 | 3.391444773 |
| **Slc12a5** | Neurogenesis | dendritic spine development | 4.64E-11 | 0.407112663 |
| **Slc1a3** | Neurogenesis | cell morphogenesis involved in neuron differentiation | 2.80E-05 | 0.596151122 |
| **Slc4a10** | Neurogenesis | pyramidal neuron development | 1.56E-06 | 0.246438574 |
| **Slc6a4** | Neurogenesis | negative regulation of neuron differentiation | 2.86E-08 | 12.91969404 |
| **Slc8a3** | Neurogenesis | oligodendrocyte differentiation | 1.11E-09 | 0.39088063 |
| **Slc9a6** | Neurogenesis | axon extension | 5.20E-05 | 0.573132256 |
|  |  | dendrite extension |  |  |
|  |  | dendritic spine development |  |  |
|  |  | neuron projection morphogenesis |  |  |
| **Slit3** | Neurogenesis | axon extension involved in axon guidance | 1.28E-10 | 2.462288827 |
| **Slitrk1** | Neurogenesis | axonogenesis | 4.26E-10 | 0.434762677 |
| **Slitrk3** | Neurogenesis | axonogenesis | 6.71E-05 | 0.456124636 |
| **Snap25** | Neurogenesis | regulation of neuron projection development | 0.00035255 | 0.39433673 |
| **Snap91** | Neurogenesis | axonogenesis | 3.48E-06 | 0.545643285 |
| **Sox10** | Neurogenesis | central nervous system myelination | 0.00033212 | 3.188327879 |
|  |  | negative regulation of Schwann cell proliferation |  |  |
|  |  | oligodendrocyte differentiation |  |  |
|  |  | positive regulation of gliogenesis |  |  |
|  |  | positive regulation of neuroblast proliferation |  |  |
| **Spock1** | Neurogenesis | negative regulation of neuron projection development | 1.48E-07 | 0.494691152 |
| **Sptbn4** |  | central nervous system projection neuron axonogenesis | 2.52E-08 | 0.438485184 |
|  |  | clustering of voltage-gated sodium channels |  |  |
| **Srcin1** | Neurogenesis | regulation of dendritic spine morphogenesis | 0.0004974 | 0.611370989 |
| **Stau2** | Neurogenesis | positive regulation of dendritic spine morphogenesis | 1.05E-05 | 0.52842477 |
| **Stmn2** | Neurogenesis | regulation of neuron projection development | 0.00061767 | 0.649256294 |
| **Stmn3** | Neurogenesis | neuron projection development | 5.10E-06 | 0.55820231 |
| **Stmn4** | Neurogenesis | neuron projection development | 0.0023824 | 0.551766587 |
| **Stxbp1** | Neurogenesis | axon target recognition | 2.23E-05 | 0.587161793 |
| **Stxbp5** | Neurogenesis | axonogenesis | 4.19E-05 | 0.567223811 |
| **Syngap1** | Neurogenesis | dendrite development | 7.41E-05 | 0.581479405 |
|  |  | negative regulation of axonogenesis |  |  |
| **Synj1** | Neurogenesis | positive regulation of gliogenesis | 4.34E-05 | 0.567251333 |
| **Syt1** | Neurogenesis | positive regulation of dendrite extension | 7.62E-07 | 0.509096129 |
| **Syt2** | Neurogenesis | positive regulation of dendrite extension | 2.17E-10 | 0.331550377 |
| **Syt3** | Neurogenesis | positive regulation of dendrite extension | 2.47E-09 | 0.436393041 |
| **Syt4** | Neurogenesis | negative regulation of short-term neuronal synaptic plasticity | 4.77E-06 | 0.558643569 |
|  |  | positive regulation of dendrite extension |  |  |
| **Tacc3** | Neurogenesis | neurogenesis | 9.42E-06 | 3.139424245 |
| **Tbc1d24** | Neurogenesis | neuron projection development | 9.11E-06 | 0.556923085 |
|  |  | positive regulation of dendrite morphogenesis |  |  |
|  |  | positive regulation of neuron migration |  |  |
| **Tcf3** | Neurogenesis | positive regulation of neuron differentiation | 4.37E-05 | 1.967986058 |
| **Tcf7l2** | Neurogenesis | oligodendrocyte development | 1.62E-05 | 2.149349359 |
|  |  | regulation of oligodendrocyte differentiation |  |  |
| **Tead3** | Neurogenesis | asymmetric neuroblast division | 4.40E-09 | 2.733595921 |
| **Tgif1** | Neurogenesis | positive regulation of neuron differentiation | 0.0012286 | 1.840707 |
| **Tgif2** | Neurogenesis | positive regulation of neuron differentiation | 0.00095116 | 2.16925552 |
| **Thbs4** | Neurogenesis | neuron projection morphogenesis | 1.45E-14 | 3.324644308 |
| **Tlx2** | Neurogenesis | negative regulation of dendrite morphogenesis | 1.16E-07 | 2.666967049 |
| **Tmem106b** | Neurogenesis | dendrite morphogenesis | 0.0034109 | 0.635546681 |
|  |  | positive regulation of dendrite development |  |  |
| **Tmem98** | Neurogenesis | negative regulation of oligodendrocyte differentiation | 9.52E-08 | 2.295804828 |
| **Tnc** | Neurogenesis | neuron projection development | 7.97E-16 | 3.274784564 |
|  |  | peripheral nervous system axon regeneration |  |  |
| **Tnik** | Neurogenesis | neuron projection morphogenesis | 0.001646 | 0.658844543 |
| **Trim46** | Neurogenesis | axonogenesis | 0.00057585 | 0.51502344 |
|  |  | negative regulation of axon extension |  |  |
|  |  | neuron migration |  |  |
| **Trim67** | Neurogenesis | positive regulation of neuron projection development | 0.004012 | 0.523078726 |
| **Trpc5** | Neurogenesis | negative regulation of dendrite morphogenesis | 0.0061511 | 0.375503953 |
|  |  | positive regulation of axon extension |  |  |
|  |  | positive regulation of neuron differentiation |  |  |
| **Tsku** | Neurogenesis | anterior commissure morphogenesis | 0.0056967 | 1.500985458 |
|  |  | corpus callosum morphogenesis |  |  |
| **Tspo** | Neurogenesis | glial cell migration | 0.0014802 | 2.255738765 |
| **Ttc3** | Neurogenesis | negative regulation of neuron differentiation | 0.0016729 | 0.578729062 |
| **Tubb2a** | Neurogenesis | neuron migration | 5.73E-07 | 0.535418911 |
| **Twist1** | Neurogenesis | neuron migration | 3.07E-20 | 7.493813118 |
| **Unc13a** | Neurogenesis | positive regulation of dendrite extension | 5.50E-05 | 0.56815245 |
| **Unc5a** | Neurogenesis | anterior/posterior axon guidance | 0.00027512 | 0.591114699 |
|  |  | axon guidance |  |  |
|  |  | neuron projection development |  |  |
| **Vax2** | Neurogenesis | axonogenesis | 6.27E-06 | 5.790147097 |
|  | Neurogenesis | neuron differentiation | 6.27E-06 | 5.790147097 |
| **Vcan** | Neurogenesis | glial cell migration | 2.40E-06 | 2.217293884 |
| **Vcl** | Neurogenesis | axon extension | 0.00027332 | 1.629342612 |
| **Vim** | Neurogenesis | negative regulation of neuron projection development | 0.0021108 | 1.767708022 |
|  |  | positive regulation of glial cell proliferation |  |  |
|  |  | regulation of axonogenesis |  |  |
|  |  | regulation of Schwann cell migration |  |  |
| **Vsx1** | Neurogenesis | neuron development | 0.00020314 | 8.067378808 |
|  |  | neuron maturation |  |  |
| **Vwc2l** | Neurogenesis | positive regulation of neuron differentiation | 0.005539 | 0.298375296 |
| **Wee1** | Neurogenesis | neuron projection morphogenesis | 1.71E-08 | 2.361003205 |
| **Wnt10a** | Neurogenesis | neuron differentiation | 0.00033353 | 4.146229575 |
| **Wnt10b** | Neurogenesis | neuron differentiation | 0.00013816 | 8.136452977 |
| **Wnt11** | Neurogenesis | neuron differentiation | 3.68E-05 | 3.328794939 |
| **Wnt2** | Neurogenesis | canonical Wnt signaling pathway involved in midbrain dopaminergic neuron differentiation | 1.63E-09 | 19.56902538 |
| **Xrcc2** | Neurogenesis | positive regulation of neurogenesis | 0.0035567 | 1.787396866 |
| **Xrcc5** | Neurogenesis | positive regulation of neurogenesis | 0.0036415 | 1.568678823 |
| **Zfp804a** | Neurogenesis | positive regulation of neuron projection development | 1.57E-07 | 0.358017 |

| **MMC vs Control E15**  **Total Genes: 87**  **Blue: Downregulated**  **Yellow Upregulated** | | | | |
| --- | --- | --- | --- | --- |
| **Gene Name** | **Annotated term** | **Context** | **p-val** | **Fold Change** |
| **Agtpbp1** | Spinal Cords & Neurons | cerebellar Purkinje cell differentiation | 5.97E-07 | 0.575800122 |
| **Ascl1** | Spinal Cords & Neurons | central nervous system neuron development | 4.19E-12 | 3.64394201 |
|  |  | commitment of neuronal cell to specific neuron type in forebrain |  |  |
|  |  | forebrain neuron differentiation |  |  |
|  |  | spinal cord association neuron differentiation |  |  |
|  |  | ventral spinal cord interneuron fate commitment |  |  |
|  | Oligodendrocytes | oligodendrocyte cell fate commitment |  |  |
|  |  | oligodendrocyte development |  |  |
| **Atp2b2** | Spinal Cords & Neurons | cerebellar granule cell differentiation | 4.66E-15 | 0.392645699 |
|  |  | cerebellar Purkinje cell differentiation |  |  |
| **Bmp4** | Oligodendrocytes | negative regulation of oligodendrocyte differentiation | 0.003137 | 1.764708629 |
| **Btg2** | Spinal Cords & Neurons | central nervous system neuron development | 3.43E-13 | 2.702697412 |
| **Cbln1** | Spinal Cords & Neurons | cerebellar granule cell differentiation | 2.30E-09 | 0.514473973 |
| **Cdh11** | Spinal Cords & Neurons | corticospinal tract morphogenesis | 0.0062794 | 1.665978751 |
| **Chd5** | Spinal Cords & Neurons | cerebral cortex neuron differentiation | 1.63E-21 | 0.317273735 |
| **Cntnap1** | Oligodendrocytes | central nervous system myelination | 2.84E-05 | 0.507866071 |
| **Dll3** | Astrocytes | negative regulation of astrocyte differentiation | 1.91E-06 | 2.302498155 |
| **Dlx2** | Spinal Cords & Neurons | cerebral cortex GABAergic interneuron differentiation | 0.0027093 | 9.212546524 |
|  |  | forebrain neuron differentiation |  |  |
|  |  | regulation of transcription from RNA polymerase II promoter involved in forebrain neuron fate commitment |  |  |
|  | Oligodendrocytes | negative regulation of oligodendrocyte differentiation |  |  |
| **Dlx5** | Spinal Cords & Neurons | olfactory bulb interneuron differentiation | 8.69E-06 | 14.23923762 |
| **Drd2** | Spinal Cords & Neurons | cerebral cortex GABAergic interneuron migration | 0.0032901 | 0.552237208 |
| **Dusp10** | Oligodendrocytes | negative regulation of oligodendrocyte differentiation | 0.0087707 | 1.831353124 |
| **Dusp15** | Oligodendrocytes | regulation of oligodendrocyte differentiation | 0.00050338 | 0.580782545 |
| **Egfr** | Astrocytes | astrocyte activation | 0.0037302 | 2.271113803 |
| **Eif2b2** | Oligodendrocytes | oligodendrocyte development | 0.00032705 | 1.542285656 |
| **Enpp2** | Oligodendrocytes | positive regulation of oligodendrocyte differentiation | 0.0006985 | 0.491648791 |
| **Ephb1** | Spinal Cords & Neurons | central nervous system projection neuron axonogenesis | 6.57E-07 | 0.592686047 |
| **Erbb2** | Oligodendrocytes | oligodendrocyte differentiation | 2.10E-06 | 2.242487588 |
| **Fgfr2** | Spinal Cords & Neurons | pyramidal neuron development | 0.00068114 | 1.994669882 |
| **Gbx2** | Spinal Cords & Neurons | forebrain neuron development | 0.00017975 | 1.919951676 |
| **Gli2** | Spinal Cords & Neurons | smoothened signaling pathway involved in spinal cord motor neuron cell fate specification | 1.94E-05 | 3.81425157 |
| **Grid2** | Spinal Cords & Neurons | cerebellar granule cell differentiation | 5.67E-05 | 0.494725442 |
| **Gsx1** | Spinal Cords & Neurons | spinal cord association neuron differentiation | 5.49E-05 | 3.35939165 |
| **Gsx2** | Spinal Cords & Neurons | olfactory bulb interneuron differentiation | 5.54E-15 | 4.876006334 |
|  |  | spinal cord association neuron differentiation |  |  |
|  | Oligodendrocytes | positive regulation of oligodendrocyte differentiation |  |  |
| **Hdac1** | Oligodendrocytes | positive regulation of oligodendrocyte differentiation | 9.29E-11 | 2.055657625 |
| **Hdac11** | Oligodendrocytes | oligodendrocyte development | 3.07E-11 | 0.393353957 |
| **Hes1** | Astrocytes | positive regulation of astrocyte differentiation | 1.11E-09 | 2.172716588 |
|  | Spinal Cords & Neurons | negative regulation of forebrain neuron differentiation |  |  |
|  | Oligodendrocytes | negative regulation of oligodendrocyte differentiation |  |  |
| **Hes5** | Astrocytes | Negative regulation of astrocyte differentiation | 1.06E-06 | 2.781571133 |
|  | Oligodendrocytes | negative regulation of oligodendrocyte differentiation |  |  |
| **Hmga2** | Astrocytes | negative regulation of astrocyte differentiation | 9.88E-13 | 3.125961479 |
| **Hoxc10** | Spinal Cords & Neurons | spinal cord motor neuron cell fate specification | 6.24E-19 | 117.7024059 |
| **Hoxd10** | Spinal Cords & Neurons | spinal cord motor neuron cell fate specification | 4.05E-22 | 4980.241544 |
| **Il34** | Oligodendrocytes | positive regulation of oligodendrocyte differentiation | 3.34E-06 | 0.117905261 |
| **Inhba** | Spinal Cords & Neurons | striatal medium spiny neuron differentiation | 0.0021405 | 3.372456887 |
| **Kndc1** | Spinal Cords & Neurons | cerebellar granule cell differentiation | 6.07E-20 | 0.285527182 |
| **Ldlr** | Astrocytes | negative regulation of astrocyte activation | 0.0049251 | 1.545999658 |
| **Mag** | Astrocytes | positive regulation of astrocyte differentiation | 2.49E-08 | 0.137766784 |
|  | Oligodendrocytes | central nervous system myelination |  |  |
| **Map2** | Spinal Cords & Neurons | central nervous system neuron development | 2.72E-07 | 0.58932689 |
| **Mdk** | Oligodendrocytes | positive regulation of oligodendrocyte differentiation | 1.04E-11 | 2.042165799 |
| **Med12** | Oligodendrocytes | oligodendrocyte development | 0.00099494 | 1.757567381 |
| **Mt3** | Astrocytes | astrocyte development | 1.72E-10 | 0.303254298 |
| **Nanos1** | Spinal Cords & Neurons | cerebellar neuron development | 3.98E-09 | 0.463615273 |
| **Neurog2** | Spinal Cords & Neurons | central nervous system neuron development | 5.97E-05 | 2.740235717 |
| **Nkx6-1** | Spinal Cords & Neurons | central nervous system neuron differentiation | 0.00019641 | 1.544371676 |
|  |  | regulation of transcription from RNA polymerase II promoter involved in spinal cord motor neuron fate specification |  |  |
|  | Oligodendrocytes | oligodendrocyte differentiation |  |  |
| **Notch1** | Astrocytes | positive regulation of astrocyte differentiation | 0.0014245 | 2.216986523 |
|  | Oligodendrocytes | negative regulation of oligodendrocyte differentiation |  |  |
| **Nrxn1** | Spinal Cords & Neurons | cerebellar granule cell differentiation | 1.22E-09 | 0.484712099 |
| **Ntrk2** | Spinal Cords & Neurons | central nervous system neuron development | 5.55E-07 | 0.579194577 |
|  | Oligodendrocytes | oligodendrocyte differentiation |  |  |
| **Ntrk3** | Astrocytes | negative regulation of astrocyte differentiation | 0.00012023 | 0.586364635 |
| **Olig1** | Oligodendrocytes | oligodendrocyte differentiation | 3.78E-33 | 0.189885283 |
| **Olig2** | Spinal Cords & Neurons | spinal cord motor neuron differentiation | 2.86E-17 | 0.22440862 |
|  | Oligodendrocytes | oligodendrocyte differentiation |  |  |
| **Omg** | Oligodendrocytes | central nervous system myelination | 5.84E-05 | 0.252980835 |
| **Pax3** | Spinal Cords & Neurons | spinal cord association neuron differentiation | 9.30E-06 | 3.798158206 |
| **Pax7** | Spinal Cords & Neurons | spinal cord association neuron differentiation | 0.0045888 | 4.658288525 |
| **Plp1** | Astrocytes | astrocyte development | 0.0051713 | 1.560317906 |
|  | Oligodendrocytes | central nervous system myelination |  |  |
| **Pou3f2** | Astrocytes | astrocyte development | 0.0005305 | 1.831518153 |
| **Pou3f4** | Spinal Cords & Neurons | forebrain neuron differentiation | 0.00053661 | 1.677508411 |
| **Prox1** | Spinal Cords & Neurons | cerebellar granule cell differentiation | 0.0013984 | 2.001386775 |
|  |  | positive regulation of forebrain neuron differentiation |  |  |
| **Ptger3** | Spinal Cords & Neurons | negative regulation of forebrain neuron differentiation | 0.00058459 | 3.000701892 |
| **Ptn** | Oligodendrocytes | positive regulation of oligodendrocyte differentiation | 3.56E-08 | 0.539382268 |
| **Ptpra** | Oligodendrocytes | positive regulation of oligodendrocyte differentiation | 0.00013235 | 0.657093238 |
| **Rac3** | Spinal Cords & Neurons | cerebral cortex GABAergic interneuron development | 7.03E-09 | 0.400757104 |
| **Robo2** | Spinal Cords & Neurons | olfactory bulb interneuron development | 1.89E-06 | 0.599441138 |
| **Ror1** | Astrocytes | astrocyte development | 0.0035956 | 3.090413747 |
| **Sall1** | Spinal Cords & Neurons | olfactory bulb interneuron differentiation | 0.0070534 | 2.012655149 |
| **Samd4b** | Spinal Cords & Neurons | cerebellar neuron development | 0.00045025 | 1.531271393 |
| **Sfrp2** | Spinal Cords & Neurons | regulation of midbrain dopaminergic neuron differentiation | 3.68E-07 | 4.167262559 |
| **Slc45a3** | Oligodendrocytes | regulation of oligodendrocyte differentiation | 0.0035871 | 2.599798842 |
| **Slc4a10** | Spinal Cords & Neurons | pyramidal neuron development | 3.33E-16 | 0.191617337 |
| **Slc8a3** | Oligodendrocytes | oligodendrocyte differentiation | 3.54E-10 | 0.421031477 |
| **Smo** | Astrocytes | astrocyte activation | 0.001969 | 1.940233115 |
|  | Spinal Cords & Neurons | central nervous system neuron differentiation |  |  |
| **Sox10** | Oligodendrocytes | central nervous system myelination | 0.00018938 | 3.704297579 |
|  |  | oligodendrocyte differentiation |  |  |
| **Sox2** | Spinal Cords & Neurons | forebrain neuron differentiation | 2.15E-07 | 1.98451986 |
| **Sox6** | Astrocytes | astrocyte differentiation | 0.0003414 | 1.851352997 |
|  | Oligodendrocytes | oligodendrocyte cell fate specification |  |  |
| **Sox9** | Astrocytes | astrocyte fate commitment | 0.0013602 | 2.806943452 |
|  | Oligodendrocytes | oligodendrocyte differentiation |  |  |
| **Sufu** | Spinal Cords & Neurons | smoothened signaling pathway involved in spinal cord motor neuron cell fate specification | 0.0057143 | 2.304094678 |
| **Tcf7l2** | Oligodendrocytes | oligodendrocyte development | 3.45E-10 | 2.83451126 |
| **Tfap2a** | Spinal Cords & Neurons | forebrain neuron development | 2.03E-16 | 2.705508924 |
| **Tgfb1** | Microglia | positive regulation of microglia differentiation | 0.0020605 | 1.550872409 |
|  | Oligodendrocytes | oligodendrocyte development |  |  |
| **Tmem98** | Oligodendrocytes | negative regulation of oligodendrocyte differentiation | 3.22E-12 | 2.655162208 |
| **Tsku** | Spinal Cords & Neurons | anterior commissure morphogenesis | 0.00054695 | 1.552399636 |
| **Tulp3** | Spinal Cords & Neurons | central nervous system neuron differentiation | 0.0042827 | 1.585876032 |
| **Unc5d** | Spinal Cords & Neurons | pyramidal neuron differentiation | 0.00059977 | 0.427293714 |
| **Uncx** | Spinal Cords & Neurons | olfactory bulb interneuron differentiation | 0.0067746 | 1.721611954 |
| **Vim** | Astrocytes | astrocyte development | 2.51E-05 | 2.05081876 |
|  |  | Bergmann glial cell differentiation |  |  |
| **Vtn** | Oligodendrocytes | oligodendrocyte differentiation | 2.02E-05 | 0.454830214 |
| **Wnt3a** | Spinal Cords & Neurons | spinal cord association neuron differentiation | 0.010362 | 5.224887594 |
| **Vehicle vs Control E15**  **Total Genes: 7**  **Blue: Downregulated**  **Yellow Upregulated** | | | | |

| **Gene Name** | **Annotated Term** | **Context** | **p-val** | **Fold Change** |
| --- | --- | --- | --- | --- |
| **Avil** | neurogenesis | positive regulation of neuron projection development | 3.04E-05 | 0.548905614 |
| **Hdac6** | neurogenesis | collateral sprouting | 8.56E-05 | 0.089529069 |
| **Hoxc10** | neurogenesis | spinal cord motor neuron cell fate specification | 1.09E-121 | 0.020838569 |
|  | central nervous system neuron differentiation |  |  |  |
| **Hoxd10** | neurogenesis | peripheral nervous system neuron development | 8.77E-120 | 0.000446826 |
|  |  | spinal cord motor neuron cell fate specification |  |  |
| **Hoxd9** | neurogenesis | peripheral nervous system neuron development | 8.09E-64 | 0.029157281 |
| **Pax7** | neurogenesis | neuron fate commitment | 6.69E-05 | 0.474736868 |
|  |  | spinal cord association neuron differentiation |  |  |
| **Ptpro** | neurogenesis | axon guidance | 1.77E-10 | 0.464515937 |
