# Supplementary material for: Premature Neural Progenitor Cell Differentiation Into Astrocytes in Retinoic Acid-Induced Spina Bifida Rat Model
Source: Front Mol Neurosci. 2022 Jun 17;15:888351. doi: 10.3389/fnmol.2022.888351 (PMC9249056; doi:10.3389/fnmol.2022.888351)
Supplement: Supplementary file 5 [file Table_4.docx]

| **MMC vs Vehicle E17**  **Total Genes: 32**  **Blue: Downregulated**  **Yellow Upregulated** | | | | | | | | |  |
| --- | --- | --- | --- | --- | --- | --- | --- | --- | --- |
| **Gene Name** | **Annotated term** | | **Context** | | **p-val** | | **Fold Change** | |  |
| **Adra2b** | Neurogenesis | | positive regulation of neuron differentiation | | 2.49E-05 | | 3.000077979 | |  |
| **Bmp6** | Neurogenesis | | positive regulation of neuron differentiation | | 1.37E-05 | | 0.407536166 | |  |
| **Cit** | Neurogenesis | | dendrite development | | 0.000202 | | 0.502348335 | |  |
|  |  |  | generation of neurons | |  |  |  |  |  |
|  |  |  | negative regulation of dendrite morphogenesis | |  |  |  |  |  |
|  |  |  | negative regulation of neuron differentiation | |  |  |  |  |  |
| **Dbx1** | Neurogenesis | | ventral spinal cord interneuron specification | | 2.97E-11 | | 14.00723215 | |  |
| **Dll3** | Neurogenesis | | regulation of neurogenesis | | 0.000199 | | 2.054802879 | |  |
| **Dmrt3** | Neurogenesis | | ventral spinal cord interneuron specification | | 6.11E-05 | | 3.360556128 | |  |
| **Epha10** | Neurogenesis | | axon guidance | | 9.85E-07 | | 0.320167772 | |  |
| **Gabra5** | Neurogenesis | | inner ear receptor cell development | | 1.58E-05 | | 0.444606209 | |  |
|  |  |  | neuron development | |  |  |  |  |  |
| **Gas1** | Neurogenesis | | axon guidance | | 0.00037 | | 2.227151964 | |  |
| **Heyl** | Neurogenesis | | positive regulation of neuron differentiation | | 0.000123 | | 2.224837551 | |  |
|  |  |  | regulation of neurogenesis | |  |  |  |  |  |
| **Hoxc10** | Neurogenesis | | spinal cord motor neuron cell fate specification | | 1.18E-11 | | 3.354272738 | |  |
| **Hoxc8** | Neurogenesis | | neuron differentiation | | 5.80E-05 | | 0.239749548 | |  |
| **Hoxd10** | Neurogenesis | | peripheral nervous system neuron development | | 2.22E-10 | | 3.156224879 | |  |
|  |  |  | spinal cord motor neuron cell fate specification | |  |  |  |  |  |
| **Hoxd9** | Neurogenesis | | peripheral nervous system neuron development | | 4.03E-06 | | 2.472893314 | |  |
| **Irx3** | Neurogenesis | | regulation of neuron differentiation | | 8.39E-07 | | 2.748415263 | |  |
| **Map1a** | Neurogenesis | | axonogenesis | | 2.14E-05 | | 0.484208396 | |  |
| **Nefh** | Neurogenesis | | axon development | | 7.90E-13 | | 0.252088114 | |  |
|  |  |  | peripheral nervous system neuron axonogenesis | |  |  |  |  |  |
| **Nefl** | Neurogenesis | | neuron projection morphogenesis | | 1.81E-05 | | 0.482399394 | |  |
|  |  |  | peripheral nervous system axon regeneration | |  |  |  |  |  |
|  |  |  | positive regulation of axonogenesis | |  |  |  |  |  |
| **Neurog1** | Neurogenesis | | neurogenesis | | 0.000193 | | 17.60605775 | |  |
|  |  |  | neuron differentiation | |  |  |  |  |  |
|  |  |  | regulation of neuron differentiation | |  |  |  |  |  |
| **Neurog2** | Neurogenesis | | axon guidance | | 1.19E-05 | | 3.951224994 | |  |
|  |  |  | central nervous system neuron development | |  |  |  |  |  |
|  |  |  | neuron migration | |  |  |  |  |  |
|  |  |  | positive regulation of neuron differentiation | |  |  |  |  |  |
| **Nrtn** | Neurogenesis | | neuron projection development | | 5.55E-05 | | 2.57629893 | |  |
| **Omg** | Neurogenesis | | central nervous system myelination | | 0.000178 | | 0.394090807 | |  |
|  |  |  | neuron projection regeneration | |  |  |  |  |  |
|  |  |  | regulation of collateral sprouting of intact axon in response to injury | |  |  |  |  |  |
| **Plppr4** | Neurogenesis | | axonogenesis | | 0.000219 | | 0.467001795 | |  |
| **Plxnd1** | Neurogenesis | | positive regulation of axonogenesis | | 1.30E-05 | | 2.258868051 | |  |
|  |  |  | semaphorin-plexin signaling pathway involved in axon guidance | |  |  |  |  |  |
| **Robo3** | Neurogenesis | | axon guidance | | 7.84E-10 | | 3.126394859 | |  |
|  |  |  | neuron migration | |  |  |  |  |  |
| **Scn1b** | Neurogenesis | | corticospinal neuron axon guidance | | 0.000163 | | 0.354240238 | |  |
|  |  |  | positive regulation of neuron projection development | |  |  |  |  |  |
| **Slc4a10** | Neurogenesis | | pyramidal neuron development | | 6.42E-05 | | 0.441443284 | |  |
| **Sox14** | Neurogenesis | | regulation of neuron migration | | 0.000337 | | 2.81532212 | |  |
| **Syt2** | Neurogenesis | | positive regulation of dendrite extension | | 4.48E-08 | | 0.338610825 | |  |
| **Thy1** | Neurogenesis | | negative regulation of axonogenesis | | 2.37E-06 | | 0.41702333 | |  |
|  |  |  | negative regulation of neuron projection regeneration | |  |  |  |  |  |
| **Tmem98** | Neurogenesis | | negative regulation of oligodendrocyte differentiation | | 0.000214 | | 2.402439333 | |  |
| **Vsx2** | Neurogenesis | | retinal bipolar neuron differentiation | | 0.000107 | | 2.28437579 | |  |
| **MMC vs Control E17**  **Total Genes: 11**  **Blue: Downregulated**  **Yellow Upregulated** | | | | | | | | |  |
| **Gene Name** | | **Annotated term** | | **Context** | | **p-val** | | **Fold Change** | |
| **Ascl1** | | Spinal Cords & Neurons | | central nervous system neuron development | | 0.00087713 | | 1.819497413 | |
|  |  |  |  | commitment of neuronal cell to specific neuron type in forebrain | |  |  |  |  |
|  |  |  |  | forebrain neuron differentiation | |  |  |  |  |
|  |  |  |  | spinal cord association neuron differentiation | |  |  |  |  |
|  |  |  |  | ventral spinal cord interneuron fate commitment | |  |  |  |  |
|  |  | Oligodendrocytes | | oligodendrocyte cell fate commitment | |  |  |  |  |
| **Atp2b2** | | Spinal Cords & Neurons | | cerebellar granule cell differentiation | | 5.40E-07 | | 0.469175635 | |
| **Cntnap1** | | Oligodendrocytes | | central nervous system myelination | | 1.96E-05 | | 0.47813834 | |
| **Dll3** | | Astrocytes | | negative regulation of astrocyte differentiation | | 6.40E-06 | | 2.170458744 | |
| **Hes1** | | Astrocytes | | positive regulation of astrocyte differentiation | | 0.00013769 | | 2.143249787 | |
|  |  | Spinal Cords & Neurons | | negative regulation of forebrain neuron differentiation | |  |  |  |  |
|  |  | Oligodendrocytes | | negative regulation of oligodendrocyte differentiation | |  |  |  |  |
| **Hoxd10** | | Spinal Cords & Neurons | | spinal cord motor neuron cell fate specification | | 7.63E-25 | | 8.122928807 | |
| **Mag** | | Astrocytes | | positive regulation of astrocyte differentiation | | 2.01E-06 | | 0.356630006 | |
|  |  | Oligodendrocytes | | central nervous system myelination | |  |  |  |  |
| **Mt3** | | Astrocytes | | astrocyte development | | 0.00010369 | | 0.533536916 | |
| **Neurog2** | | Spinal Cords & Neurons | | central nervous system neuron development | | 1.43E-07 | | 5.202842374 | |
| **Rac3** | | Spinal Cords & Neurons | | cerebral cortex GABAergic interneuron development | | 0.00048296 | | 0.594459323 | |
| **Slc4a10** | | Spinal Cords & Neurons | | pyramidal neuron development | | 4.69E-07 | | 0.399702915 | |
| **Vehicle vs Control E17**  **Total Genes: 0**  **Blue: Downregulated**  **Yellow Upregulated** | | | | | | | | | |
