# Supplementary material for: Premature Neural Progenitor Cell Differentiation Into Astrocytes in Retinoic Acid-Induced Spina Bifida Rat Model
Source: Front Mol Neurosci. 2022 Jun 17;15:888351. doi: 10.3389/fnmol.2022.888351 (PMC9249056; doi:10.3389/fnmol.2022.888351)
Supplement: Supplementary file 6 [file Table_5.docx]

| **MMC vs Vehicle E20**  **Total Genes: 12**  **Blue: Downregulated**  **Yellow: Upregulated** | | | | | | | | | |
| --- | --- | --- | --- | --- | --- | --- | --- | --- | --- |
| **Gene Name** | **Annotated term** | | **Context** | | | **p-val** | **Fold Change** | | |
| **Chat** | Neurogenesis | | dendrite development | | | 0.0097245 | 0.166858837 | | |
|  |  |  | neuron differentiation | | |  |  |  |  |
| **Gpr37l1** | Neurogenesis | | negative regulation of neuron differentiation | | | 0.034949 | 0.289332441 | | |
| **Grin2a** | Neurogenesis | | neurogenesis | | | 0.024263 | 0.276394344 | | |
| **Hdac6** | Neurogenesis | | dendritic spine morphogenesis | | | 3.37E-05 | 0.05472713 | | |
|  |  |  | collateral sprouting | | |  |  |  |  |
| **Kcnj10** | Neurogenesis | | central nervous system myelination | | | 0.018803 | 0.270574915 | | |
|  |  |  | oligodendrocyte development | | |  |  |  |  |
| **Nefh** | Neurogenesis | | axon development | | | 0.034804 | 0.286062046 | | |
|  |  |  | peripheral nervous system neuron axonogenesis | | |  |  |  |  |
| **Nkx6-2** | Neurogenesis | | central nervous system myelination | | | 0.0040191 | 0.162882103 | | |
|  |  |  | oligodendrocyte differentiation | | |  |  |  |  |
|  |  |  | positive regulation of glial cell differentiation | | |  |  |  |  |
|  |  |  | regulation of transcription from RNA polymerase II promoter involved in spinal cord motor neuron fate specification | | |  |  |  |  |
| **Ntrk1** | Neurogenesis | | axonogenesis involved in innervation | | | 0.016679 | 13.20305608 | | |
|  |  |  | neuron development | | |  |  |  |  |
|  |  |  | neuron projection development | | |  |  |  |  |
| **Olig2** | Neurogenesis | | negative regulation of neuron differentiation | | | 0.011883 | 0.275762846 | | |
|  |  |  | neuron differentiation | | |  |  |  |  |
|  |  |  | neuron fate commitment | | |  |  |  |  |
|  |  |  | oligodendrocyte differentiation | | |  |  |  |  |
|  |  |  | spinal cord oligodendrocyte cell fate specification | | |  |  |  |  |
| **Plp1** | Neurogenesis | | axon development | | | 0.023847 | 0.315278785 | | |
|  |  |  | central nervous system myelination | | |  |  |  |  |
|  |  |  | glial cell differentiation | | |  |  |  |  |
|  |  |  | neuron projection development | | |  |  |  |  |
| **Pou4f3** | Neurogenesis | | axon extension | | | 0.018837 | 4.249520317 | | |
| **Prdm12** | Neurogenesis | | neurogenesis | | | 0.03299 | 14.67715052 | | |
|  |  |  | neuron projection development | | |  |  |  |  |
| **MMC vs Control E20**  **Total Genes: 0**  **Blue: Downregulated**  **Yellow: Upregulated** | | | | | | | | | |
| **Vehicle vs Control E20**  **Total Genes: 1**  **Blue: Downregulated**  **Yellow: Upregulated** | | | | | | | | | |
| **Gene Name** | | **Annotated term** | | **Context** | **p-val** | | | **Fold Change** |  |
| **Hdac6** | | neurogenesis | | collateral sprouting | 3.33E-09 | | | 0.141718499 |  |
|  |  |  |  | dendritic spine morphogenesis |  |  |  |  |  |
